# Building Consensus on the Relevant Criteria to Screen for Depressive Symptoms Among Near-Centenarians and Centenarians: Modified e-Delphi Study

**DOI:** 10.2196/64352

**Published:** 2025-03-05

**Authors:** Carla Gomes da Rocha, Armin von Gunten, Pierre Vandel, Daniela S Jopp, Olga Ribeiro, Henk Verloo

**Affiliations:** 1 School of Health Sciences University of Applied Sciences and Arts Western Switzerland (HES-SO) Sion Switzerland; 2 Service of Old Age Psychiatry Lausanne University Hospital and University of Lausanne Prilly-Lausanne Switzerland; 3 Institute of Biomedical Sciences Abel Salazar University of Porto Porto Portugal; 4 Institute of Psychology University of Lausanne Lausanne Switzerland; 5 Swiss National Centre of Competence in Research LIVES University of Lausanne Lausanne Switzerland; 6 Nursing School of Porto (ESEP) and RISE-Health Porto Portugal

**Keywords:** centenarians, near-centenarians, depressive symptoms, depression diagnosis, screening, assessment, e-Delphi technique, web-based survey

## Abstract

**Background:**

The number of centenarians worldwide is expected to increase dramatically, reaching 3.4 million by 2050 and >25 million by 2100. Despite these projections, depression remains a prevalent yet underdiagnosed and undertreated condition among this population that carries significant health risks.

**Objective:**

This study aimed to identify and achieve consensus on the most representative signs and symptoms of depression in near-centenarians and centenarians (aged ≥95 years) through an e-Delphi study with an international and interdisciplinary panel of experts. Ultimately, the outcomes of this study might help create a screening instrument that is specifically designed for this unique population.

**Methods:**

A modified e-Delphi study was carried out to achieve expert consensus on depressive symptoms in near-centenarians and centenarians. A panel of 28 international experts was recruited. Consensus was defined as 70% agreement on the relevance of each item. Data were collected through a web-based questionnaire over 3 rounds. Experts rated 104 items that were divided into 24 dimensions and 80 criteria to identify the most representative signs and symptoms of depression in this age group.

**Results:**

The panel consisted of experts from various countries, including physicians with experience in old age psychiatry or geriatrics as well as nurses and psychologists. The response rate remained consistent over the rounds (20/28, 71% to 21/28, 75%). In total, 4 new dimensions and 8 new criteria were proposed by the experts, and consensus was reached on 86% (24/28) of the dimensions and 80% (70/88) of the criteria. The most consensual potentially relevant dimensions were *lack of hope* (21/21, 100%), *loss of interest* (27/28, 96%), *lack of reactivity to pleasant events* (27/28, 96%), *depressed mood* (26/28, 93%), and *previous episodes of depression or diagnosed depression* (19/21, 90%). In addition, the most consensual potentially relevant criteria were *despondency, gloom, and despair* (25/25, 100%); *depressed* (27/27, 100%); *lack of reactivity to pleasant events or circumstances* (28/28, 100%); *suicidal ideation* (28/28, 100%); *suicide attempt(s)* (28/28, 100%); *ruminations* (27/28, 96%); *recurrent thoughts of death or suicide* (27/28, 96%); *feelings of worthlessness* (25/26, 96%); *critical life events* (20/21, 95%); *anhedonia* (20/21, 95%); *loss of interest in activities* (26/28, 93%); *loss of pleasure in activities* (26/28, 93%); and *sadness* (24/26, 92%). Moreover, when assessing depression in very old age, the duration, number, frequency, and severity of signs and symptoms should also be considered, as evidenced by the high expert agreement.

**Conclusions:**

The classification of most elements as *relevant* highlights the importance of a multidimensional approach for optimal depression screening among individuals of very old age. This study offers a first step toward improving depression assessment in near-centenarians and centenarians. The development of a more adapted screening tool could improve early detection and intervention, enhancing the quality of mental health care for this population.

## Introduction

### Background

The centenarian population is growing significantly and is projected to increase sharply in the coming decades. As of 2024, there were approximately 722,000 centenarians worldwide, with Japan, the United States, and China having the highest numbers [1]. This figure is expected to rise to 3.4 million by 2050 and exceed 25 million by 2100 [2,3]. In Europe, notable examples include France, Italy, and Greece, each with >20 centenarians per 100,000 inhabitants registered already in 2011. Portugal also stands out with a density of 14.4 centenarians per 100,000 inhabitants that same year, ranking it among the top 15 in Europe [4]. In Switzerland, the centenarian population more than doubled from 787 in 2000 to 1948 in 2022, reaching a noteworthy prevalence of 22.1 per 100,000 inhabitants [5].

Amid extreme longevity, individuals’ health experiences vary significantly, from robust health to substantial declines [6-8]. Depression, a prevalent mood disorder among older adults [9], emerges as a major public health concern [10]. The *International Classification of Diseases, 11th Revision* characterizes a depressive episode as “a period of depressed mood or diminished interest in activities occurring most of the day, nearly every day during a period lasting at least two weeks accompanied by other symptoms such as difficulty concentrating, feelings of worthlessness or excessive or inappropriate guilt, hopelessness, recurrent thoughts of death or suicide, changes in appetite or sleep, psychomotor agitation or retardation, and reduced energy or fatigue” [6]. However, this definition broadly encompasses the general population and may not fully address the possible difference and heterogeneity of depressive symptomatology in older adults, potentially leading to diagnostic imprecision.

Accurate diagnosis is crucial as depression should not be misconstrued as an inherent aspect of aging even in very advanced age [11,12]. A systematic literature review reported substantial variations in depression prevalence among near-centenarians and centenarians across countries, such as 12.8% in Italy, 13.5% in Australia, 20% in the United States, and 29% in Mexico [13]. These differences may reflect cultural, health care, and socioeconomic influences as well as methodological variations in study conduct [13,14]. Interestingly, comparisons between centenarian cohorts and younger groups have shown inconsistent findings, with half of the studies reporting higher rates of depressive symptoms among centenarians [13]. This highlights the diversity of aging experiences and suggests that mental health may not necessarily worsen with age. Particularly noteworthy are the findings from Sweden, where near-centenarians and centenarians exhibited almost double the depression rates of older adults aged 85 years at 32.3% versus 16.8%, respectively. These statistics highlight the need not only for careful mental health evaluations among individuals of very old age but also for prompt discussion on tailored approaches to support mental well-being in this population [13,15].

Depression can lead to severe physical, cognitive, and psychological effects. Physically, it can cause malnutrition, falls, delirium, functional decline, and increased mortality [13,16-20]. Cognitively, depression has been connected to cognitive decline and dementia [21-25]. Psychologically, it has been associated with anxiety and suicidal ideation [26,27]. Furthermore, depression places a significant burden on caregivers and health care systems [16-18,28,29], increasing costs and resource demands. These consequences underscore the critical need for early detection and intervention. Therefore, proactive measures such as regular mental health screenings are essential to prevent the severe impact of depression on individuals’ quality of life and alleviate its wider societal effects [11]; however, these are not part of the current practice in most countries worldwide.

Addressing this gap in care is difficult as depression in older adults frequently remains underdiagnosed and undertreated, in part because its manifestations are often misinterpreted as natural aspects of aging [18,19,30,31]. Studies have estimated that approximately half of depression cases are not identified by frontline health care providers [16,18], and of those detected, approximately half do not receive adequate treatment [16]. The challenge in detecting late-life depression (LLD) stems from its sometimes subtle, hidden, or atypical signs and symptoms [30,32]. Physical manifestations such as weight loss, psychomotor retardation, and exhaustion, as well as emotional manifestations such as loss of interest in activities, heightened anxiety, or irritability, might overshadow the typical sadness often associated with depression [9,18]. This complexity requires greater clinical vigilance. Moreover, the possible overlap of symptoms between depression and dementia adds another layer of diagnostic difficulty as both conditions can present with symptoms such as memory impairment, psychomotor retardation, and reduced motivation [9]. Beyond dementia, several other medical conditions can mimic or exacerbate depressive symptoms. Chronic pain, thyroid dysfunction, Parkinson disease, stroke, and medication side effects—particularly in polymedicated individuals—can contribute to *depression-like* symptoms, including fatigue, apathy, functional dependence, memory impairment, and disturbances in mood and sleep [33-38]. These overlapping symptoms underscore the need for a comprehensive assessment to distinguish LLD from other conditions, aiming for an accurate differential diagnosis. The wide range of signs and symptoms associated with LLD can extend beyond the conventional diagnostic criteria for depressive disorders. Symptom clusters not fully meeting the diagnostic criteria for depression may still carry significant clinical importance due to their link to reduced quality of life and increased disability [9,18].

### Objectives

Given the prevalence of depression among individuals of very old age, better detection of early signs and symptoms is crucial. However, despite the availability of various screening tools, to the best of our knowledge, none have been specifically validated for near-centenarians and centenarians (aged ≥95 years). Therefore, our study aimed to compile a comprehensive range of potentially relevant depressive features that have been documented in scientific literature. These features were then submitted to an international panel of experts to achieve consensus on the most representative signs and symptoms of depression in individuals of very old age. The outcomes of this approach are 2-fold. First, by systematically examining and integrating diverse diagnostic criteria and screening tools, this study aimed to contribute to the ongoing debate about the complexities and challenges of effectively diagnosing depression in individuals of very old age. Second, achieving expert consensus is central to developing a new screening instrument specifically tailored to this unique population. This effort might be an important first step toward early detection and effective intervention, which might lead to better outcomes and enhanced general well-being for this age group, and seeks to contribute to promoting mental health and supporting optimal care for individuals of very old age.

## Methods

### Study Design

We conducted a modified e-Delphi study to systematically combine expert opinions and achieve an informed group consensus on the symptomatology of depression in very old age. The Delphi technique is an established approach in which a panel of experts is asked to provide their opinions on a given issue over the course of several rounds [39]. In each round, the questions are informed by the findings of the previous one, allowing the study to evolve over time based on the collected data [39].

Given the prevalence of depression among older adults and the complexities of its diagnosis, the Delphi technique is particularly useful for gathering expert consensus on this issue. In our study, we used an e-Delphi approach to involve experts from different locations and facilitate efficient data collection through electronic communication methods. To adapt the traditional Delphi approach to the specific requirements of our study, we implemented several modifications (referred to as a *modified e-Delphi study*). To keep the focus on the items rather than on the degree of agreement, experts were informed at the start of each new round about which items had reached consensus without being given the precise consensus rates. In addition, for items on which consensus had already been reached, experts were unable to review their previous responses. This decision was made to avoid revisiting settled issues and manage the large number of items efficiently, ensuring the stability of agreed-upon elements. An additional aspect of our modified methodology was a final evaluation of open issues by the steering committee. This last step took place to discuss the items that had not achieved consensus over the e-Delphi rounds, ensuring a thorough examination of these elements.

The materials presented to the e-Delphi panel were based on a comprehensive analysis of the existing literature on depression in very old age (personal communication by Gomes da Rocha et al, 2023). This ensured that the expert panel’s input was based on a thorough understanding of the relevant literature, thereby enhancing the validity of the final consensus, which may be seen as a lever to encourage scientific debate and foster new developments toward an effective diagnosis of depression in very old age [40].

This study adhered to the Conducting and Reporting Delphi Studies guidelines [41,42].

### Steering Committee

The steering committee for this e-Delphi study based in Western Switzerland comprised 4 members: 2 physicians who are old age psychiatrists and senior researchers (AvG and PV), 1 clinical nurse specialist in geriatric care and a senior researcher skilled in the e-Delphi technique (HV), and 1 clinical nurse specialist in geriatric care who is also a junior researcher (CGdR). The committee was responsible for overseeing the study design, ensuring ethical compliance, and guiding the iterative process of questionnaire refinement and consensus building. Meetings were held monthly, with methodological decisions made based on discussion. The committee played a crucial role in maintaining the study’s rigor, managing data collection and analysis, and addressing any lack of consensus among panelists.

### Selection of Experts

In the Delphi approach, nonprobability sampling methods are used through the purposive selection of an expert panel. However, clear selection criteria were applied to limit researcher bias based on a five-step procedure [43]:

1. *Identifying the most appropriate categories of experts for the panel:* we identified experts from 3 main professional categories—physicians, nurses, and psychologists—each with a minimum of 5 years of professional experience and competencies in LLD. These professionals were selected for their specific work experience or contact with individuals of very old age in clinical, research, or teaching domains within the fields of old age psychiatry or geriatrics. In addition, our recruitment strategy aimed for a broad geographical representation by including experts from various continents (ie, Asia, Australia, Europe, and North America) to enrich the panel with diverse perspectives and, thereby, enhance the global view on depression screening among individuals of very old age.
2. *Identifying experts:* names were compiled from various sources, including previous research involvement, publications on the subject, professional email lists, professional associations or societies, and boards of professional organizations. This method aimed to create a list of potential panelists recognized for their expertise and contributions to the field that was as comprehensive as possible.
3. *Contacting some readily reachable experts from each professional category to nominate other experts:* initial contact was made with experts who were easily accessible. These experts were then asked to nominate additional professionals whom they believed could contribute valuable insights to the study, thereby using a snowball sampling technique to ensure a wide and diverse pool of expertise.
4. *Creating sublists for each professional category and ranking experts based on specific criteria:* experts were organized into sublists by professional category and ranked according to their representation of professional role or specialty and their field of practice. This was a strategic approach intended to foster a diverse and balanced panel that reflects a broad spectrum of experiences and viewpoints, contributing to the robustness and credibility of the consensus process.
5. *Inviting the experts.*

The Delphi method offers no consensus on an ideal sample size as the focus is on qualitative depth and the use of expert judgment for a comprehensive exploration of specific topics rather than on statistical representativeness [44]. Usually, a panel comprises 7 to 15 experts to facilitate effective information processing and decision-making; however, some studies may involve up to 30 experts for broader perspectives [44]. For our study, we aimed to assemble a convenience sample of at least 20 experts from various countries.

### Recruitment Procedure

On the basis of the predefined selection of potential panelists, we emailed invitation letters to a total of 84 experts. These letters outlined the study’s objectives, described the e-Delphi context, detailed the questionnaire (eg, language, type of questions, and estimated completion time), and clarified what was expected from the participants, ensuring that they understood the significance of their contributions to the study. Interested parties could access the questionnaire through a direct URL to the survey. Anticipating a 25% to 30% response rate for the first-round questionnaire, we expected approximately 20 to 25 experts to participate. We aimed to retain these experts across all subsequent rounds, planning for a maximum of 4 rounds.

### Data Collection Procedures

The SurveyMonkey tool (SurveyMonkey Inc) was used as the survey software as it facilitates secure and efficient data collection. In accordance with literature recommendations [43], each e-Delphi round remained open for a minimum of 4 weeks, during which up to 3 automatic reminder emails were dispatched to nonrespondents. At the start of each new round, experts were provided access to the overall results from the previous round, including a list of items for which consensus was either *achieved* (≥70% agreement) or *not achieved* (<70% agreement) [43]. The items that did not reach consensus were integrated into the current round for further deliberation, along with any new items that emerged from panel suggestions through open-ended questions.

### Measures

The questionnaire was divided into 3 sections.

#### e-Delphi Section

This section aimed to achieve consensus on the topic under study and consisted of 104 items. These were divided into 24 dimensions (eg, *impaired concentration* and *lack of hope*) and 80 criteria (eg, *impaired ability to think or concentrate* and *hopelessness*), which experts rated according to their relevance using a Likert scale from 1 (*Not relevant*) to 4 (*Very relevant*). Experts also had the possibility to comment on their answers and suggest additional items that they considered relevant to the topic for subsequent rounds.

In addition to rating the relevance of each item, experts were asked to indicate the most suitable type of assessment for each criterion: self-assessment, hetero-assessment, or both. This was intended to identify preferred methods of assessing potential depressive signs and symptoms. The aim was not to achieve a stringent consensus of 70% as with the relevance ratings but to capture a broad spectrum of expert opinions on assessment approaches.

The initial selection of 104 items was based on a comprehensive review of existing instruments widely used in research to screen for and evaluate depressive signs and symptoms among near-centenarians and centenarians [13] supplemented by additional insights from recent analysis (personal communication by Gomes da Rocha et al, 2023). We started with all items available in these measures to be as comprehensive as possible. These instruments included self-assessment tools such as the Geriatric Depression Scale–30 [45], the Center for Epidemiologic Studies Depression Scale [46], the depression-related items from the Brief Symptom Inventory [47], and the Hospital Anxiety and Depression Scale [48], as well as the Montgomery-Åsberg Depression Rating Scale [49], which is administered by a professional rater. Furthermore, given the significant prevalence of neurocognitive disorders in our aging population and the need for reliable assessment of depressive symptoms in individuals with cognitive impairments, we also considered instruments specifically designed for such assessments: the Cornell Scale for Depression in Dementia [50,51] and the depression section of the Neuropsychiatric Inventory [52,53]. In addition, we included the *International Classification of Diseases, 11th Revision* and the *Diagnostic and Statistical Manual of Mental Disorders, Fifth Edition* diagnostic criteria to anchor our selections in the gold standards for depression diagnosis [6,54]. To complete the pool of items, the Depression Rating Scale from the Resident Assessment Instrument was also retained given its widespread use among adults of old and very old age in diverse settings, such as private homes (Resident Assessment Instrument–Home Care) or long-term care facilities (Resident Assessment Instrument–Long-Term Care Facilities) [17,55-57]. After compiling all items from the selected instruments, we checked for overlap and excluded identical items, which led to the final selection of 104 items. For instance, items such as “Do you feel that your situation is hopeless?” from the Geriatric Depression Scale–30 and “Feeling hopeless about the future” from the Brief Symptom Inventory necessitated thematic grouping under a unified dimension (*Lack of Hope* in this example). This process enabled us to construct more general items designated as *dimensions* that reflected the essence of the criteria within each group, thereby streamlining the questionnaire without compromising the depth of depressive symptom screening. The list of all selected instruments and original items can be found in Multimedia Appendix 1.

In summary, this strategic selection of items aimed to cover a broad spectrum of relevant criteria for the screening of depressive signs and symptoms, ensuring the questionnaire’s comprehensive scope and its applicability to adults of very old age with varying levels of cognitive functioning.

#### Sociodemographic and Professional Characteristics

This section included questions designed to capture the sociodemographic and professional characteristics of the panelists in round 1, including age, gender, nationality, profession, years of professional experience, and their country and field of practice. In subsequent rounds, only essential information (age, gender, nationality, and profession) was collected to ensure continuity and to accurately link responses from the same panelists across all rounds.

#### Contact With Individuals of Very Old Age

Although this could be considered part of professional characterization, this section specifically gathered information on the frequency of the panelists’ experiences or interactions with individuals of very old age (aged ≥85 and ≥95 years). It was specific to the first round, aiming to inform the context of their responses. While this study’s target population was defined as near-centenarians and centenarians (≥95 years), there is only a limited number of experts focusing on this exact population. However, most experts working with individuals aged ≥85 years have regular contact with near-centenarians and centenarians. Thus, these experts were also invited provided that they felt confident in drawing on their experiences and perceptions relevant to the population aged ≥95 years.

### Pretest

Before launching round 1, the questionnaire was pretested with a small purposive sample of 5 professionals (nurses, psychologists, and a physician) to verify its clarity and comprehensibility, with only a minor adjustment made based on 1 expert’s suggestion to include specific examples for certain items. Despite the positive outcomes of the pretest, experts were invited to rate the questionnaire at the end of round 1 for comprehensiveness, readability, and clarity.

Overall, 93% (26/28) of the experts participating in round 1 rated the questionnaire as *Comprehensive* or *Very comprehensive*, and 86% (24/28) found it *Easy* or *Very easy* to read and understand. Due to this positive feedback, no further structural adjustments were implemented before proceeding to the subsequent rounds.

### Data Analysis

Data collected via SurveyMonkey were transferred to a Microsoft Excel spreadsheet (Microsoft Corp) and examined for any potential errors or missing data. They were then imported into the SPSS Statistics software (version 29.0; IBM Corp) for analysis [58]. Descriptive statistics were computed to characterize the panel from a sociodemographic and professional perspective using means and SDs as well as medians and interquartile ranges for quantitative variables and frequency distributions and percentages for qualitative variables. The level of agreement among experts required to achieve consensus on a given item was set at 70% for each round. This threshold is commonly used in Delphi studies [59-61] as it allows for the inclusion of minority viewpoints in the early rounds, ensuring that diverse perspectives are considered before achieving final consensus. This is particularly relevant in the context of depressive symptom screening given the challenges associated with detecting LLD, as previously mentioned. Hence, this consensus rate is considered acceptable for preserving methodological rigor, as supported by the literature [43], which indicates that such a threshold aligns with norms for ensuring reliability and validity in the context of an iterative, consensus-building approach. The consensus rate for each item was calculated using a 4-level Likert scale (1=*Not relevant*, 2=*Somewhat relevant*, 3=*Relevant*, and 4=*Very relevant*). In the first step, these responses were recoded into 2 levels: values of 1 and 2 were recoded as “0” (*Overall not relevant*), and values of 3 and 4 were recoded as “1” (*Overall relevant*). Next, we aggregated the recoded responses for each item to determine consensus, which was achieved when the percentage of experts identifying an item as *Overall relevant* or *Overall not relevant* met or exceeded the predefined threshold of 70%.

After the first e-Delphi round, items that did not reach the consensus level were carried forward to the subsequent round. At the beginning of each subsequent round, experts were given the opportunity to review the general outcomes from the previous round (ie, *consensus achieved* or *consensus not achieved* for each evaluated item). Items that achieved consensus were not included again in subsequent rounds. After conducting 3 rounds with the participating panel and observing only limited changes in the consensus rate after the third round, we decided to proceed with the last round, which involved a final evaluation of unresolved aspects by the steering committee. This last step aimed to gain a deeper understanding of the reasoning behind the outcomes and further refine the consensus process. Items that still did not achieve consensus after the 3 e-Delphi rounds were thoroughly examined during this phase.

### Ethical Considerations

The Ethics Commission on Human Research of the Canton of Vaud was consulted and confirmed that this project fell outside the scope of the Swiss Human Research Act (Req-2023-00844; Multimedia Appendix 2) as the study did not involve the collection of health-related personal data or biological material.

All participants received detailed information about the study, including its aim and relevance, what to expect, a description of the questionnaire, instructions for participation, and contact information for the main researcher, along with the invitation email. The questionnaires for all rounds (1, 2, and 3) included an introductory section emphasizing the voluntary nature of participation in the study, as well as specific information regarding confidentiality, data protection, risks, and benefits and a statement on ethical considerations. Participants were explicitly informed that, by answering the questionnaire, they were implicitly providing consent to take part in the study. They did not receive any compensation for their participation. Each participant received a survey link for accessing the questionnaire, and their IP addresses were not recorded to ensure confidentiality. The collected data were deidentified, with no personal identifiers retained. Responses stored on SurveyMonkey were deleted after transferring the data to the Microsoft Excel spreadsheet. All data are securely stored on a password-protected institutional server to comply with Swiss data protection regulations and will be definitively deleted after 5 years.

## Results

### Expert Participation and Sociodemographic and Professional Characteristics

A total of 84 experts were invited to this modified e-Delphi study. Of these 84 experts, 28 (33%) participated in the first round, forming the baseline panel. This group (n=28) was consequently invited to the second and third rounds, for which the participation rates were 75% (21/28) and 71% (20/28), respectively.

The panel exhibited sociodemographic and professional diversity, including different nationalities, professions, and fields of practice. The average age ranged slightly between 44.1 (SD 10.6) and 46.7 (SD 11.4) years over the rounds. A majority of women participated in the first and second rounds (17/28, 61% and 13/21, 62%, respectively), whereas men were more prevalent in the third round (11/20, 55%). Swiss and Portuguese nationalities were the most represented (12/20, 60% to 18/28, 64%), with the nursing profession being predominant (9/20, 45% to 12/21, 57%), followed by the medical profession (5/21, 24% to 8/20, 40%). The average professional experience was 19.4 (SD 8-9) years, with nearly 80% (22/28, 79%) of the experts being engaged in purely clinical activities or a combination of clinical and academic activities, among others (eg, service organization). More than 80% (23/28, 82%) reported having regular or very regular contact (daily, weekly, or monthly) with individuals aged ≥85 years, and 75% (21/28) reported having regular or very regular contact with individuals aged ≥95 years (Table 1).

**Table 1:** Sociodemographic and professional characteristics of the experts from the panel.

				Round 1 (n=28)		Round 2 (n=21)		Round 3 (n=20)
**Age (y)**								
	Values, mean (SD)		44.1 (10.6)		45.2 (11.6)		46.7 (11.4)	
	Values, median (IQR)		39.5 (18.8)		39.0 (22.0)		44.0 (22.8)	
**Gender, n (%)**								
	Men		11 (39)		8 (38)		11 (55)	
	Women		17 (61)		13 (62)		9 (45)	
**Nationality, n (%)**								
	Portuguese		9 (32)		6 (29)		4 (20)	
	Swiss		9 (32)		7 (33)		8 (40)	
	French		3 (11)		2 (10)		3 (15)	
	Swiss and Portuguese		2 (7)		2 (10)		1 (5)	
	Australian		1 (4)		1 (5)		1 (5)	
	Belgian		1 (4)		1 (5)		1 (5)	
	German		1 (4)		1 (5)		0 (0)	
	Italian		1 (4)		0 (0)		1 (5)	
	Norwegian		1 (4)		1 (5)		1 (5)	
**Profession, n (%)**								
	Nurse		15 (54)		12 (57)		9 (45)	
	Physician		8 (29)		5 (24)		8 (40)	
	Psychologist		4 (14)		4 (19)		3 (15)	
	Other: gerontologist		1 (4)		0 (0)		0 (0)	
**Professional experience (y; n=27)**								
	Values, mean (SD)		19.4 (8.9)		—^a^		—	
	Values, median (IQR)		17.0 (11.0		—		—	
**Country of professional practice, n (%)**								
	Switzerland		15 (54)		—		—	
	Portugal		7 (25)		—		—	
	France		3 (11)		—		—	
	Australia		1 (4)		—		—	
	Norway		1 (4)		—		—	
	Portugal, United Kingdom, Saudi Arabia, and UAE^b^		1 (4)		—		—	
**Field of professional activity, n (%)**								
	**Clinical activity**							
		Inpatient care only	6 (21)		—		—	
		Outpatient care only	2 (7)		—		—	
		Long-term care only	2 (7)		—		—	
	**Academic activity**							
		Teaching only	1 (4)		—		—	
		Research only	1 (4)		—		—	
		Teaching and research	3 (11)		—		—	
		Clinical activity^c^ and academic activity^d^	9 (32)		—		—	
		Clinical activity^c^, academic activity^d^, and other (eg, quality improvement or service organization)	3 (11)		—		—	
		Management activity	1 (4)		—		—	
**Contact with individuals aged ≥85 years, n (%)**								
	Daily		10 (36)		—		—	
	Weekly		9 (32)		—		—	
	Monthly		4 (14)		—		—	
	Yearly		4 (14)		—		—	
	Previously but not currently		1 (4)		—		—	
**Contact with individuals aged ≥95 years, n (%)**								
	Daily		4 (14)		—		—	
	Weekly		8 (29)		—		—	
	Monthly		9 (32)		—		—	
	Yearly (or less^e^)		6 (21)		—		—	
	Previously but not currently		1 (4)		—		—	

^a^Not applicable.

^b^UAE: United Arab Emirates.

^c^*Clinical activity* includes inpatient, outpatient, or long-term care settings.

^d^*Academic activity* includes teaching or research roles.

^e^One expert commented the following: “Not yearly, but once in a while.”

### Questionnaire Completion Times

The completion dates and the mean time taken for participants to complete the questionnaires in each round are summarized in Table 2. Globally, the e-Delphi rounds took place between August 2023 and February 2024, with an overall weighted mean completion time of 16 minutes and 40 seconds (SD 11 min and 27 s).

**Table 2:** Summary of overall consensus achievements across e-Delphi rounds regarding potential dimensions and criteria for depression screening.

	Questionnaire completion dates	Questionnaire completion time, mean (SD)	Dimensions analyzed	Consensus achieved (dimensions), n (%)	Consensus not achieved (dimensions), n (%)	New dimensions proposed	Criteria analyzed	Consensus achieved (criteria), n (%)	Consensus not achieved (criteria), n (%)	New criteria proposed
Round 1	August 15, 2023, to October 1, 2023	24 min, 51 s (10 min, 51 s)	24	13 (54)^a^	11 (46)^a^	4	80	49 (61)^b^	31 (39)^b^	8
Round 2	October 26, 2023, to December 12, 2023	15 min, 31 s (8 min, 18 s)	15	10 (67)^c^	5 (33)^c^	0	39	12 (31)^d^	27 (69)^d^	0
Round 3	January 31, 2024, to February 29, 2024	6 min, 26 s (3 min, 55 s)	5	1 (20)^e^	4 (80)^e^	0	27	9 (33)^f^	18 (67)^f^	0

^a^n=24.

^b^n=80.

^c^n=15.

^d^n=39.

^e^n=5.

^f^n=27.

### Overall Consensus Achievements

A consensus threshold of ≥70% was established for both dimensions and criteria, as previously described.

#### Depression Screening: Potentially Relevant Dimensions

In round 1, experts assessed 24 dimensions, reaching consensus on 13 (54%). In addition, 4 new dimensions were suggested by the experts and incorporated in round 2: *underlying cognitive disorder* (dimension 25), *previous episodes of depression or diagnosed depression* (dimension 26), *personal care (eg, poor hygiene)* (dimension 27), and *language/speech (eg, poor speech)* (dimension 28). Thus, 15 dimensions (n=11, 73% without consensus and n=4, 27% new proposals) were reassessed in round 2, with consensus achieved on 10 (67%). In round 3, a total of 5 dimensions were revisited, with consensus reached on only 1 (20%; Table 2).

Table 3 summarizes the consensus rates for the 28 dimensions assessed across the 3 e-Delphi rounds. A total of 14% (4/28) of the dimensions failed to reach the ≥70% consensus threshold: memory problems (dimension 21), mood-congruent delusions (dimension 22), reduced adaptation capacity (dimension 24), and language/speech (eg, poor speech) (dimension 28)—the only newly suggested dimension without consensus. Of the dimensions on which consensus was achieved, all were considered relevant for screening for depression in individuals aged ≥95 years except for increased appetite (dimension 8), which was deemed not relevant. Notably, several relevant dimensions reached ≥90% consensus: lack of hope (dimension 20; 21/21, 100%), loss of interest (dimension 4; 27/28, 96%), lack of reactivity to pleasant events (dimension 5; 27/28, 96%), depressed mood (dimension 1; 26/28, 93%), and previous episodes of depression or diagnosed depression (dimension 26; 19/21, 90%).

Figure S1 in Multimedia Appendix 3 provides an overview of the consensus rates for the potentially relevant dimensions for depression screening.

**Table 3:** Consensus level on potential dimensions for depression screening^a^.

	Round 1 (n=28)			Round 2 (n=21)			Round 3 (n=20)		
	Level of consensus at ≥70%	Result	Participants, n (%)	Level of consensus at ≥70%	Result	Participants, n (%)	Level of consensus at ≥70%	Result	Participants, n (%)
Depressed mood	Achieved	Relevant	26 (93)^b^	—^c^	—	—	—	—	—
Anxiety	Achieved	Relevant	24 (86)^b^	—	—	—	—	—	—
Irritability	Not achieved	N/A^d^	N/A	Achieved	Relevant	17 (81)^e^	—	—	—
Loss of interest	Achieved	Relevant	27 (96)^b^	—	—	—	—	—	—
Lack of reactivity to pleasant events	Achieved	Relevant	27 (96)^b^	—	—	—	—	—	—
Psychomotor changes	Not achieved	N/A	N/A	Achieved	Relevant	16 (76)^e^	—	—	—
Appetite loss	Achieved	Relevant	19 (70)^f^	—	—	—	—	—	—
Increased appetite	Not achieved	N/A	N/A	Achieved	Not relevant	16 (76)^e^	—	—	—
Reduced sleep or insomnia	Achieved	Relevant	19 (73)^g^	—	—	—	—	—	—
Hypersomnia	Not achieved	N/A	N/A	Achieved	Relevant	16 (76)^e^	—	—	—
Lack of energy	Achieved	Relevant	21 (78)^f^	—	—	—	—	—	—
Death wishes	Achieved	Relevant	22 (79)^b^	—	—	—	—	—	—
Suicidal thoughts	Achieved	Relevant	24 (86)^b^	—	—	—	—	—	—
Poor self-esteem	Achieved	Relevant	23 (82)^b^	—	—	—	—	—	—
Pessimism	Achieved	Relevant	20 (74)^f^	—	—	—	—	—	—
Low life satisfaction	Achieved	Relevant	20 (71)^b^	—	—	—	—	—	—
Impaired concentration	Not achieved	N/A	N/A	Not achieved	N/A	N/A	Achieved	Relevant	14 (70)^h^
Impaired decision-making capacity	Not achieved	N/A	N/A	Achieved	Relevant	15 (71)^e^	—	—	—
Negative feelings	Achieved	Relevant	24 (86)^b^	—	—	—	—	—	—
Lack of hope	Not achieved	N/A	N/A	Achieved	Relevant	21 (100)^e^	—	—	—
Memory problems	Not achieved	N/A	N/A	Not achieved	N/A	N/A	Not achieved	N/A	N/A
Mood-congruent delusions	Not achieved	N/A	N/A	Not achieved	N/A	N/A	Not achieved	N/A	N/A
Repercussions of functional dependence	Not achieved	N/A	N/A	Achieved	Relevant	16 (76)^e^	—	—	—
Reduced adaptation capacity	Not achieved	N/A	N/A	Not achieved	N/A	N/A	Not achieved	N/A	N/A
Underlying cognitive disorder (added in round 2)	N/A	N/A	N/A	Achieved	Relevant	15 (71)^e^	—	—	—
Previous episodes of depression or diagnosed depression (added in round 2)	N/A	N/A	N/A	Achieved	Relevant	19 (90)^e^	—	—	—
Personal care (eg, poor hygiene; added in round 2)	N/A	N/A	N/A	Achieved	Relevant	16 (76)^e^	—	—	—
Language/speech (eg, poor speech; added in round 2)	N/A	N/A	N/A	Not achieved	N/A	N/A	Not achieved	N/A	N/A

aThis list includes 4 dimensions suggested by the expert participants in round 1 and incorporated into the subsequent rounds: underlying cognitive disorder, previous episodes of depression or diagnosed depression, personal care (eg, poor hygiene), and language/speech (eg, poor speech).

^b^n=28.

^c^Data are not available for the corresponding entries.

^d^N/A: not applicable.

^e^n=21.

^f^n=27.

^g^n=26.

^h^n=20.

#### Depression Screening: Potentially Relevant Criteria

In round 1, a total of 80 criteria were assessed, with consensus reached on 49 (61%). In addition, 8 new criteria emerged from suggestions by the panel and were incorporated in round 2: *critical life events (eg, loss of a child or another loved one)* (criterion 81), *emotional indifference* (criterion 82), *anhedonia* (criterion 83), *afraid of being alone* (criterion 84), *resurfacing of “old wounds” (eg, long-standing conflicts with children)* (criterion 85), *feeling of accomplishment throughout life* (criterion 86), *closeness with family and significant others* (criterion 87), and *subjective health* (criterion 88). As a result, 39 criteria (n=31, 79% without consensus and n=8, 21% new proposals) were reassessed in round 2, with consensus achieved on 12 (31%). In round 3, a total of 27 criteria were revisited, with consensus reached on 9 (33%; Table 2).

The consensus rates for the 88 criteria assessed across the 3 e-Delphi rounds are presented in Multimedia Appendix 4. In total, 20% (18/88) of the criteria failed to reach the ≥70% consensus threshold: *easily annoyed* (criterion 20); *short-tempered* (criterion 21); *staying home instead of going out and doing new things* (criterion 26); *psychomotor agitation* (criterion 29); *significant unintentional weight gain (more than 5% in a month)* (criterion 33); *restless sleep* (criterion 35); *full of energy* (criterion 42); *feels as good as other people* (criterion 51); *pessimism* (criterion 52); *thinks most people are better off than him/her* (criterion 56); *finding life exciting, wonderful, and enjoyable* (criterion 58); *getting bored often* (criterion 62); *feeling fearful* (criterion 64); *feeling that people are unfriendly or dislike him/her* (criterion 66); *memory problems* (criterion 69); *the mind is as clear as it used to be* (criterion 70); *having a sense of direction and purpose in life* (criterion 79); and *afraid of being alone* (criterion 84)—the only newly suggested criterion without consensus. Of the criteria on which consensus was achieved, all were considered *relevant* for screening for depression in individuals aged ≥95 years except for *hard to get started on new projects* (criterion 28), *fear of dying* (criterion 75), and *sufficient financial resources* (criterion 77), which were considered *not relevant*. Notably, several *relevant* criteria reached a ≥90% consensus: *despondency, gloom, and despair* (criterion 5; 25/25, 100%); *depressed* (criterion 6; 27/27, 100%); *lack of reactivity to pleasant events or circumstances* (criterion 25; 28/28, 100%); *suicidal ideation* (criterion 45; 28/28, 100%); *suicide attempt(s)* (criterion 46; 28/28, 100%); *ruminations* (criterion 14; 27/28, 96%); *recurrent thoughts of death or suicide* (criterion 43; 27/28, 96%); *feelings of worthlessness* (criterion 47; 25/26, 96%); *critical life events* (criterion 81; 20/21, 95%); *anhedonia* (criterion 83; 20/21, 95%); *loss of interest in activities* (criterion 23; 26/28, 93%); *loss of pleasure in activities* (criterion 24; 26/28, 93%); and *sadness* (criterion 4; 24/26, 92%).

Figure S2 in Multimedia Appendix 3 provides an overview of the consensus rates for the potentially relevant criteria for depression screening.

### Signs and Symptoms of Depression: Duration, Number, Frequency, and Severity

In round 1, experts (n=28) were asked about the importance of considering the duration of depressive signs or symptoms in addition to their presence. It is important to note that, for the purpose of this assessment, they were instructed not to consider *suicidal thoughts or ideation* as these require immediate attention and, thus, were deliberately excluded from the duration criteria. All participants (28/28, 100%) agreed on its importance. When specifically asked about the minimum duration for considering these signs or symptoms as clinically significant, 25 experts provided their insights—21 (84%) suggested a range from *a few days* (1 to 3) to *4 weeks*, with *2 weeks* being the most cited (n=9, 36%).

In addition, 7% (2/28) of the experts suggested differentiating the duration of signs or symptoms between recurrent episodes in chronic depression and new-onset cases. For this reason, a question addressing this specificity was introduced in round 2 (n=21), with 62% (13/21) supporting the distinction. For recurrent episodes, most experts favored a minimum duration of *2 weeks* (9/13, 69%), whereas for a new onset, opinions varied, with “4 weeks” (5/13, 38%) being the most cited.

In round 2 (n=21), 62% (13/21) emphasized the importance of considering the number of signs or symptoms during screening. Of the 12 experts who specified a threshold, 7 (58%) favored a minimum of 3 symptoms.

Regarding sign or symptom frequency, all experts who provided an opinion in round 2 (20/20, 100%) considered it relevant. Of these 20 experts, 19 (95%) specified a particular frequency, of whom 9 (47%) suggested a frequency of *several times per day*, 3 (16%) suggested a frequency of *nearly daily*, and 6 (32%) suggested a frequency of *several times per week*.

For severity, 90% (18/20) considered it an important factor. Moreover, on a severity scale from 0 to 10 (with 10 being the most severe), the average threshold for clinical concern was 4.9 (SD 1.4), with a median of 5.0 (IQR 2.0).

Further details regarding the results described previously can be found in Multimedia Appendix 5.

### Steering Committee’s Decision on Dimensions and Criteria Lacking Consensus After 3 e-Delphi Rounds

The steering committee conducted 2 additional rounds of discussion to decide on the relevance of the 4 dimensions and 18 criteria that did not reach a consensus of ≥70% among the panelists during the 3 e-Delphi rounds.

In the first round of discussion, committee members reviewed the panelists’ comments and agreement levels for each dimension and criterion, analyzing potential reasons for the lack of consensus and considering the clinical implications. Each member shared their perspectives drawing on their clinical expertise. In the second round, the committee revisited the dimensions and criteria after reflecting on the insights from the first round, aiming to resolve any remaining uncertainties and reach a collective decision. Consensus was prioritized, with majority voting used when necessary.

A summary of the committee’s final determinations is provided in Multimedia Appendix 6, where the rationale behind the inclusion or exclusion of each dimension and criterion is detailed.

Table 4 presents an overview of the consensus levels reached in each e-Delphi round and the final decisions made by the steering committee.

**Table 4:** Steering committee’s decision on the dimensions and criteria lacking consensus after 3 e-Delphi rounds.

				Round 1 (n=28), n (%)		Round 2 (n=21), n (%)		Round 3 (n=20), n (%)		Steering committee final decision
**Dimensions**										
	**Memory problems**									Not relevant
		Not relevant	15 (56)^a^		11 (52)^b^		9 (45)^c^			
		Relevant	12 (44)^a^		10 (48)^b^		11 (55)^c^			
	**Mood-congruent delusions**									Relevant
		Not relevant	12 (44)^a^		9 (43)^b^		9 (45)^c^			
		Relevant	15 (56)^a^		12 (57)^b^		11 (55)^c^			
	**Reduced adaptation capacity**									Relevant
		Not relevant	12 (43)^d^		7 (33)^b^		8 (40)^c^			
		Relevant	16 (57)^d^		14 (67)^b^		12 (60)^c^			
	**Language or speech (eg, poor speech; added in round 2)**									Not relevant
		Not relevant	—^e^		8 (38)^b^		12 (60)^c^			
		Relevant	—		13 (62)^b^		8 (40)^c^			
**Criteria**										
	**Easily annoyed**									Not relevant
		Not relevant	11 (39)^d^		12 (57)^b^		11 (55)^c^			
		Relevant	17 (61)^d^		9 (43)^b^		9 (45)^c^			
	**Short-tempered**									Relevant
		Not relevant	11 (41)^a^		11 (58)^f^		10 (50)^c^			
		Relevant	16 (59)^a^		8 (42)^f^		10 (50)^c^			
	**Staying home instead of going out and doing new things**									Not relevant
		Not relevant	11 (39)^d^		9 (43)^b^		12 (60)^c^			
		Relevant	17 (61)^d^		12 (57)^b^		8 (40)^c^			
	**Psychomotor agitation**									Not relevant
		Not relevant	11 (41)^a^		8 (38)^b^		9 (45)^c^			
		Relevant	16 (59)^a^		13 (62)^b^		11 (55)^c^			
	**Significant unintentional weight gain (more than 5% in a month)**									Not relevant
		Not relevant	18 (67)^a^		14 (67)^b^		11 (55)^c^			
		Relevant	9 (33)^a^		7 (33)^b^		9 (45)^c^			
	**Restless sleep**									Relevant
		Not relevant	10 (38)^g^		8 (40)^c^		8 (40)^c^			
		Relevant	16 (62)^g^		12 (60)^c^		12 (60)^c^			
	**Full of energy**									Not relevant
		Not relevant	18 (67)^a^		12 (57)^b^		12 (60)^c^			
		Relevant	9 (33)^a^		9 (43)^b^		8 (40)^c^			
	**Feels as good as other people**									Not relevant
		Not relevant	14 (52)^a^		13 (65)^c^		8 (40)^c^			
		Relevant	13 (48)^a^		7 (35)^c^		12 (60)^c^			
	**Pessimism**									Relevant
		Not relevant	9 (32)^d^		9 (45)^c^		8 (40)^c^			
		Relevant	19 (68)^d^		11 (55)^c^		12 (60)^c^			
	**Thinks most people are better off than him or her**									Not relevant
		Not relevant	10 (37)^a^		9 (43)^b^		7 (35)^c^			
		Relevant	17 (63)^a^		12 (57)^b^		13 (65)^c^			
	**Finding life exciting, wonderful, and enjoyable**									Not relevant
		Not relevant	10 (37)^a^		9 (45)^c^		9 (45)^c^			
		Relevant	17 (63)^a^		11 (55)^c^		11 (55)^c^			
	**Getting bored often**									Not relevant
		Not relevant	11 (41)^a^		12 (57)^b^		7 (37)^f^			
		Relevant	16 (59)^a^		9 (43)^b^		12 (63)^f^			
	**Feeling fearful**									Relevant
		Not relevant	9 (33)^a^		11 (52)^b^		10 (50)^c^			
		Relevant	18 (67)^a^		10 (48)^b^		10 (50)^c^			
	**Feeling that people are unfriendly or dislike him or her**									Not relevant
		Not relevant	15 (56)^a^		13 (62)^b^		7 (35)^c^			
		Relevant	12 (44)^a^		8 (38)^b^		13 (65)^c^			
	**Memory problems**									Not relevant
		Not relevant	14 (50)^d^		12 (57)^b^		9 (45)^c^			
		Relevant	14 (50)^d^		9 (43)^b^		11 (55)^c^			
	**The mind is as clear as it used to be**									Not relevant
		Not relevant	14 (52)^a^		11 (52)^b^		10 (50)^c^			
		Relevant	13 (48)^a^		10 (48)^b^		10 (50)^c^			
	**Having a sense of direction and purpose in life**									Relevant
		Not relevant	11 (41)^a^		7 (33)^b^		9 (45)^c^			
		Relevant	16 (59)^a^		14 (67)^b^		11 (55)^c^			
	**Afraid of being alone (added in round 2)**									Relevant
		Not relevant	—		9 (43)^b^		8 (40)^c^			
		Relevant	—		12 (57)^b^		12 (60)^c^			

^a^n=27.

^b^n=21.

^c^n=20.

^d^n=28.

^e^Not applicable.

^f^n=19.

^g^n=26.

### Types of Assessment for Potential Depression Criteria: Self-Assessment, Hetero-Assessment, or Both?

In addition to assessing the relevance of each criterion, the panel provided opinions on the preferred assessment method (self-assessment, hetero-assessment, or both). Rather than seeking strict consensus, the goal was to identify general trends. Using a 50% threshold as an example, 35% (28/80) of the criteria were deemed suitable for *self-assessment* only, 4% (3/80) of the criteria were deemed suitable for *hetero-assessment* only, and 40% (32/80) of the criteria were deemed suitable for both methods. Detailed information is available in Multimedia Appendix 7, whereas Figure S3 in Multimedia Appendix 3 provides an overview of the consensus rates for these assessment methods.

## Discussion

### Principal Findings

This modified e-Delphi study aimed to identify and achieve consensus on the most representative signs and symptoms of depression in very old age, focusing on near-centenarians and centenarians.

Our results revealed that most of the items proposed—86% (24/28) of the dimensions and 80% (70/88) of the criteria—which had been assembled based on a literature review, were considered *relevant* for depression screening in very old age. This highlights the importance of a multidimensional approach and acknowledges the complexity of accurately diagnosing depression in this population.

### Most Consensual Potentially Relevant Depressive Signs and Symptoms

Focusing on items with the highest consensus rates (≥90%), some overlap between dimensions and criteria was observed, which was expected as dimensions grouped related criteria.

For example, the *depressed mood* dimension was widely considered *relevant*, aligning with criteria such as *depressed*; *sadness*; and *despondency, gloom, and despair*, which reflect the emotional core of depressive disorders [6,54]. Similarly, *loss of interest in activities*, *loss of pleasure in activities*, *lack of reactivity to pleasant events or circumstances*, and *anhedonia* also achieved broad consensus as critical indicators of depression [6,54,62-64]. Collectively, these signs and symptoms highlight the significant impact of depression on a person’s capacity for joy and engagement with life, underscoring their relevance in depression screening.

Criteria such as *ruminations* and *feelings of worthlessness* equally achieved a strong consensus due to their role in the negative cognitive and emotional patterns of depression. Previous research has shown that *ruminations* exacerbate psychopathology, including depression, by intensifying and extending negative mood states, thus interfering with problem-solving and perpetuating physiological stress responses [65]. Similarly, *feelings of worthlessness* are strongly linked to self-blame, lack of hope, and an increased suicide risk even after depressive episodes remit [66,67]. Building on this, the *lack of* *hope* dimension achieved unanimous consensus (21/21, 100%) as a relevant element, reflecting its importance as a significant predictor of psychological well-being [68]. Within this broader dimension, the *hopelessness* and *hopeful about the future* criteria also showed high consensus (24/27, 89% and 16/21, 76%, respectively), emphasizing their important role in depression screening in individuals of very old age whether as a negative indicator or a positive resource.

Furthermore, *previous episodes of depression or diagnosed depression* were also identified as relevant, highlighting the importance of considering an individual’s psychiatric history as past experiences with depression increase the risk of recurrence [69]. In addition, *critical life events* perceived as negative were recognized for their significant association with higher levels of depressive symptoms in a recent meta-analysis [70].

Finally, the strong agreement on severe criteria such as *recurrent thoughts of death or suicide*, *suicidal ideation*, and past *suicide attempt(s)* underscores the critical need to address these factors due to their immediate risk to individuals’ safety [26].

### Most Consensual Potentially Relevant Depressive Signs and Symptoms in the Context of Old Age

Having established the most consensual depressive features, it is essential to explore how these characteristics manifest specifically in older adults as unique age-related factors may shape their expression and impact.

As individuals age, they may face different challenges, some of which are particularly relevant to LLD. These include agism; loss of income; the death of loved ones; and the risk of isolation, functional limitations, multimorbidity, cognitive decline, and institutionalization [71]. Such multidimensional challenges can impact well-being and mental health, contributing to reduced aging satisfaction and increased loneliness, including near-centenarians and centenarians [71,72]. While the cumulative effect of these difficulties can increase the risk of LLD, it is important to note that experiencing them does not necessarily lead to the development of a depressive state [72].

The dimensions and criteria that achieved high consensus, as previously discussed, clearly represent central elements in depression screening across all age groups, aligning with current diagnostic standards [6,54]. Our findings confirm their importance for accurate diagnosis in individuals of very old age, underscoring their transversality across generations and relevance even in the final stages of life. While depression in very old age may have unique aspects, some of the core signs and symptoms of the disorder remain consistent and should not be overlooked, including in near-centenarians and centenarians. However, these signs and symptoms may carry additional significance due to the unique psychosocial and physical challenges faced by these individuals [72].

For instance, *loss of interest in activities*, *loss of pleasure in activities*, *lack of reactivity to pleasant events or circumstances*, and *anhedonia* may highlight a deeper disengagement from life that can be difficult to detect in older adults. These symptoms are often masked by typical aging challenges such as physical decline, social isolation, or even cognitive issues, complexifying their identification. In addition, *feelings of worthlessness* and the negative impact of *critical life events* may be particularly relevant in late-life contexts, where significant life changes and losses are common [67,73]. As highlighted in a meta-synthesis [74], older adults often experience declines in physical and cognitive abilities, leading to feelings of disconnection and a perception of being unneeded by their communities. This sense of being unneeded can foster feelings of isolation and insignificance, thereby triggering or exacerbating depressive symptoms. Moreover, societal changes and loss of social roles further deepen these feelings of worthlessness, underscoring the importance of addressing these issues in older populations [74].

While the high consensus on many classic criteria suggests their continued relevance across the life cycle, our findings also point to unique aspects of depression in very old age that may not be fully captured by current approaches. For example, the unanimous agreement on the relevance of *lack of* *hope* highlights its particular significance in this age group. Unlike other factors typically associated with normal aging processes, low levels of hope have been identified as a significant risk factor in older adults, reflecting a deeper sense of despair with potentially serious consequences. A systematic review [75] underscored that lack of hope is closely linked to negative outcomes in older populations, including an increased risk of depression and poorer overall health outcomes.

Moreover, the strong agreement on severe symptoms such as *recurrent thoughts of death or suicide*, *suicidal ideation*, and past *suicide attempt(s)* underscores the importance of also addressing these factors in very old age, where such topics often remain taboo [76]. Reluctance to discuss or acknowledge these symptoms may lead to underdiagnosis and inadequate treatment, emphasizing the need for sensitive and proactive approaches in this age group. Recent qualitative research [76] has revealed that suicidal thoughts in later life are often subtly expressed and normalized in everyday conversations, making them difficult to detect. However, the extent to which this applies to centenarians is unclear. Previous research suggests that only a minority of centenarians express a wish to die [77,78], making explicit suicidal thoughts likely rare and not always linked to depressive states. Nonetheless, feelings of life being *completed and no longer worth living* reflect a deeper disconnection from life, characterized by aching loneliness, feelings of not mattering, and a physical and mental tiredness [79]. Creating safe spaces for informal—yet meaningful—discussions is essential, alongside strategies for timely recognition and management of these symptoms [76].

### Potentially Not Relevant Depressive Signs and Symptoms in the Context of Old Age

The *increased appetite* dimension may have been considered *not relevant* as it can result from situational factors such as attending a day center or moving to a nursing home, where tailored meals and social dining often enhance the willingness to eat, contributing to an increased appetite. Similarly, the *significant unintentional weight gain* criterion was likely deemed *not relevant* for related reasons as previous research has found no association between weight gain and incident depressive symptoms in older adults [80].

Certain cognitive dimensions such as *memory problems* and *language/speech (eg, poor speech)* were also classified as *not relevant*, likely due to their overlap with nondepressive phenomena commonly observed in older adults. This highlights the need for tailored screening protocols that can effectively differentiate between depression and cognitive impairment, conditions that often coexist in individuals of very old age and require distinct therapeutic strategies [81,82]. The criterion *the mind is as clear as it used to be* was likely deemed *not relevant* for the same reasons.

Criteria such as *hard to get started on new projects*; *staying home instead of going out and doing new things*; *finding life exciting, wonderful, and enjoyable*; *easily annoyed*; and *getting bored often* may be more closely related to motivational factors, social involvement, social support, or underlying health conditions rather than directly indicating a depressive state [83,84]. Similarly, criteria such as *thinks most people are better off than him/her*, *feeling that people are unfriendly or dislike him/her*, and *feels as good as other people* may reflect personality traits, which are relatively stable over the life span and may not necessarily reflect a change in mental health status [85].

Symptoms such as *psychomotor agitation* illustrate the challenge in differentiating between depression-related symptoms and those arising from other geriatric conditions such as dementia or delirium [86], suggesting that certain depressive features may be less applicable or not sufficient for individuals of very old age [87]. In addition, the *full of energy* criterion, typically seen as a positive indicator, was not deemed relevant, possibly because it is rare for a near-centenarian or centenarian without depression to report feeling full of energy. This likely reflects their overall physical condition rather than a depressive state, and the absence of this criterion may often be confounded with physical exhaustion linked to frailty [88].

The *fear of dying* criterion was also classified as *not relevant*. Although it might be assumed that individuals in the final stage of life would fear death, previous research suggests that death anxiety is generally low among older adults, who are more often concerned with the dying process rather than death itself [89]. Similarly, *sufficient financial resources* was deemed *not relevant*. While financial difficulties can impact mental health, their link to psychological distress in older adults appears to be more influenced by underlying factors such as mastery and self-esteem rather than acting as a direct indicator of depression [90].

### The Specificity of Near-Centenarians and Centenarians

Research on near-centenarians and centenarians has predominantly focused on physical, cognitive, and social health, with less attention paid to psychiatric aspects [13]. Studies on depression in this population often rely on overall assessment scores from standardized instruments rather than detailed analysis of individual items [72,87,91-94]. This gap, alongside the variety of existing tools for screening for depression in very old age, also motivated this study.

Notably, one study conducted a detailed item-level analysis using a 14-item version of the Geriatric Depression Scale in Portuguese centenarians [88] and found that 51.4% reported *feelings of worthlessness*, supporting the relevance of this specific criterion in our e-Delphi study. In addition, only 12.3% reported having more *memory problems*, 43.3% expressed a preference for *staying home instead of going out and doing new things*, 39.8% reported *getting bored often*, and 33.3% agreed with the statement *thinks most people are better off than they are* [88]. Globally, these findings align with our results, where these criteria were considered not relevant in this age group.

While these findings suggest reduced presence of symptoms established for younger populations, it remains unclear whether depression in near-centenarians and centenarians significantly differs from that in other older individuals, such as octogenarians; the key features of depression may not vary meaningfully. Therefore, it is worth questioning whether the development of a tool specifically designed for very old age, including the near-centenarian and centenarian population, is truly necessary. Nevertheless, psychological resources such as resilience appear to be particularly strong in near-centenarians and centenarians [77,95]. These protective characteristics may lead traditional depression screening tools to overlook subtle signs of depression that are masked by these strengths. A tool specifically designed for this age group could better capture the subtle differences and specific needs, enhancing the accuracy and effectiveness of depression screening.

### Duration, Number, Frequency, and Severity of Depressive Signs and Symptoms and Type of Assessment

Our findings emphasize the importance of considering the duration, number, frequency, and severity of depressive signs and symptoms in near-centenarians and centenarians. Notably, experts differentiated between chronic and new-onset depression, advocating for a nuanced approach that may significantly impact clinical assessments and interventions. This aligns with existing literature documenting distinct clinical profiles for persistent and nonchronic depressive disorders in adults aged 18 to 79 years [96,97], with persistent cases often involving earlier onset, more severe symptoms, and greater treatment resistance [96]. While these findings are based on younger individuals, they reinforce the necessity of a personalized life span approach that includes rigorous screening and comprehensive clinical strategies for the oldest age groups.

Furthermore, experts in our study highlighted the potential value of combining self- and hetero-assessment techniques to screen for depressive signs and symptoms in very old age. However, this approach may require further consideration. Cognitive limitations at this age may affect the reliability of self-reports, complicating the integration with caregiver observations. Developing strategies to reconcile these differing perspectives—such as guidelines for interpreting discrepancies—could ensure that assessments are accurate and sensitive to the unique challenges faced by individuals of very old age. Flexible, combined approaches remain relevant as research suggests that they enhance diagnostic accuracy and patient engagement [98].

### Further Considerations

Given the findings of our study, a more comprehensive approach to depression screening in near-centenarians and centenarians may be advantageous. The high consensus among experts regarding certain depressive features highlights their relevance and significance for this particular age group. Importantly, the diagnosis of LLD in adults of very old age can be particularly complex due to overlapping symptoms with other mental or somatic conditions, as previously outlined. This overlap necessitates a differential diagnostic approach that integrates a broader perspective. This emphasizes the need for screening tools that are both comprehensive and sensitive to the unique experiences and needs of individuals of very old age. Implementing such tailored screening methods could contribute to improved mental health outcomes within this population.

Our work extends existing literature by providing a comprehensive overview of a wider range of depressive features, including considerations of their duration, number, frequency, and severity—factors that are less considered in standard depression screenings. This highlights the complexity of diagnosing depression in adults of very old age and confirms the necessity of incorporating these specific aspects into the screening process.

While the e-Delphi method was valuable for identifying key elements in detecting depressive signs and symptoms in those of very old age, a separate validation study is necessary to confirm whether the extended screening process would be more effective compared to current tools. Nonetheless, this study hopefully represents a meaningful step forward in improving the detection of depressive signs and symptoms in this population.

### Limitations

We acknowledge several limitations. First, despite the international and professional diversity of the expert panel, it may not have captured the full spectrum of clinical practices or cultural perceptions related to aging and depression. The relative overrepresentation of nurses among the panelists in rounds 1 and 2 may have introduced a bias toward nursing-specific priorities, such as a stronger focus on the practical applicability of screening criteria in daily care. However, this also reflects their pivotal role in direct care for individuals of very old age, making their insights particularly relevant for developing pragmatic screening criteria. Given this, a *groupthink* effect in which the dominant profession influenced the consensus cannot be excluded. In addition, our panel lacked experts from Asian countries despite notable recruitment efforts even though these regions have some of the highest proportions of centenarians worldwide. This absence may have introduced a bias toward Western perspectives. At the same time, the concept of depression in very old age may be less clearly defined in some Asian cultures due to cultural background. Second, the selection of a 70% consensus threshold may have influenced the interpretation of the results as it reflects a compromise between inclusivity and stringency, subtly shaping the scope of the consensus achieved. Third, the structured nature of the e-Delphi process may have limited the exploration of nuanced symptoms critical to understanding depression in individuals of very old age, a population whose cognitive decline may complicate symptom presentation. Fourth, some overlap between dimensions and criteria was observed, which may be perceived as a limitation to some extent. However, the steering committee was aware of this overlap and intentionally decided to retain it to confirm certain trends in consensus on specific items.

Despite these challenges, this study’s insights have the potential to stimulate discussions on improving mental health strategies for older populations, especially those of very old age. The diverse and interdisciplinary composition of the panel further contributes to the generalizability of the findings, offering valuable insights to health care professionals and researchers worldwide.

Future research should aim to develop and validate a screening tool specifically designed for depressive signs and symptoms in very old age, including near-centenarians and centenarians. A validation study comparing the extended method with established tools or a *gold standard* diagnostic reference is crucial to determining its efficacy. In addition, it is important to assess the tool’s effectiveness across different cultural contexts to ensure its broad applicability. The incorporation of Patient-Reported Experience Measures in future developments would also be beneficial in capturing the lived experiences and perceptions of this age group.

### Conclusions

This expert consensus could significantly contribute to enhancing early detection and intervention for depression in individuals of very old age, notably by informing the creation of a new screening tool. The findings have the potential to improve diagnostic accuracy and, consequently, allow for the personalization of treatment plans tailored to the unique needs of individuals of very old age.

In conclusion, this study used expert consensus to establish a foundational step toward enhancing the detection and treatment of depression among individuals of very old age, including near-centenarians and centenarians. This study emphasizes the need for cautious, multidimensional, and multidisciplinary approaches to depression screening in this age group, advocating for the continuous refinement of screening tools to address the complex nature of very old age effectively.

## Appendix

Multimedia Appendix 1 Selected instruments and their respective items.

Multimedia Appendix 2 Decision of the ethics committee.

Multimedia Appendix 3 Graphical overview of the consensus rates (dimensions, criteria, and type of assessment).

Multimedia Appendix 4 Consensus level on potential criteria for depression screening.

Multimedia Appendix 5 Aspects of signs and symptoms of depression (duration, number, frequency, and severity).

Multimedia Appendix 6 The steering committee's final determinations.

Multimedia Appendix 7 Findings on the preferred assessment methods for depression screening criteria.

## Abbreviations

LLD: late-life depression
