# Supplementary material for: Building Consensus on the Relevant Criteria to Screen for Depressive Symptoms Among Near-Centenarians and Centenarians: Modified e-Delphi Study
Source: JMIR Aging. 2025 Mar 5;8:e64352. doi: 10.2196/64352 (PMC11923476; doi:10.2196/64352)
Supplement: Multimedia Appendix 1 [file aging_v8i1e64352_app1.docx]

DIAGNOSTIC CRITERIA

- **International Classification of Diseases 11^th^ Revision (ICD-11) criteria** [1]

“*A depressive episode is characterised by a period of depressed mood or diminished interest in activities occurring most of the day, nearly every day during a period lasting at least two weeks accompanied by other symptoms such as difficulty concentrating, feelings of worthlessness or excessive or inappropriate guilt, hopelessness, recurrent thoughts of death or suicide, changes in appetite or sleep, psychomotor agitation or retardation, and reduced energy or fatigue.*”

1. Depressed mood [icd_1]
2. Diminished interest in activities [icd_2]
3. Concentration difficulties [icd_3]
4. Feelings of worthlessness [icd_4]
5. Excessive or inappropriate guilt [icd_5]
6. Hopelessness [icd_6]
7. Recurrent thoughts of death or suicide [icd_7]
8. Changes in appetite [icd_8]
9. Changes in sleep [icd_9]
10. Psychomotor agitation or retardation [icd_10]
11. Reduced energy [icd_11]
12. Fatigue [icd_12]

- **Diagnostic and Statistical Manual of Mental Disorders, Fifth Edition (DSM-5) criteria** [2]

Five or more of the following A criteria (at least one includes A1 or A2):

A1. Depressed mood (indicated by subjective report or observation by others) [dsm_a1]

A2. Loss of interest or pleasure in almost all activities (indicated by subjective report or observation by others) [dsm_a2]

A3. Significant (more than 5% in a month) unintentional weight loss/gain or decrease/increase in appetite [dsm_a3]

A4. Sleep disturbance (insomnia or hypersomnia) [dsm_a4]

A5. Psychomotor changes (agitation or retardation) severe enough to be observable by others [dsm_a5]

A6. Tiredness, fatigue, or low energy, or decreased efficiency with which routine tasks are completed [dsm_a6]

A7. A sense of worthlessness or excessive, inappropriate, or delusional guilt (not merely self-reproach or guilt about being sick) [dsm_a7]

A8. Impaired ability to think, concentrate, or make decisions (indicated by subjective report or observation by others) [dsm_a8]

A9. Recurrent thoughts of death (not just fear of dying), suicidal ideation, or suicide attempts [dsm_a9]

- Symptoms must persist most of the day, daily, for at least 2 weeks in a row, excluding A3 and A9.

INSTRUMENTS:

- **Geriatric Depression Scale – 30 items (GDS-30)** [3]

1. Are you basically satisfied with your life? [gds_1]; 2. Have you dropped many of your activities and interests? [gds_2]; 3. Do you feel that your life is empty? [gds_3]; 4. Do you often get bored? [gds_4]; 5. Are you hopeful about the future? [gds_5]; 6. Are you bothered by thoughts you can’t get out of your head? [gds_6]; 7. Are you in good spirits most of the time? [gds_7]; 8. Are you afraid that something bad is going to happen to you? [gds_8]; 9. Do you feel happy most of the time? [gds_9]; 10. Do you often feel helpless? [gds_10]; 11. Do you often get restless and fidgety? [gds_11]; 12. Do you prefer to stay at home, rather than going out and doing new things? [gds_12]; 13. Do you frequently worry about the future? [gds_13]; 14. Do you feel you have more problems with memory than most? [gds_14]; 15. Do you think it is wonderful to be alive now? [gds_15]; 16. Do you often feel downhearted and blue? [gds_16]; 17. Do you feel pretty worthless the way you are now? [gds_17]; 18. Do you worry a lot about the past? [gds_18]; 19. Do you find life very exciting? [gds_19]; 20. Is it hard for you to get started on new projects? [gds_20]; 21. Do you feel full of energy? [gds_21]; 22. Do you feel that your situation is hopeless? [gds_22]; 23. Do you think that most people are better off than you are? [gds_23]; 24. Do you frequently get upset over little things? [gds_24]; 25. Do you frequently feel like crying? [gds_25]; 26. Do you have trouble concentrating? [gds_26]; 27. Do you enjoy getting up in the morning? [gds_27]; 28. Do you prefer to avoid social gatherings? [gds_28]; 29. Is it easy for you to make decisions? [gds_29]; 30. Is your mind as clear as it used to be? [gds_30]

- **Center for Epidemiologic Studies Depression Scale (CES-D)** [4]

1. I was bothered by things that usually don’t bother me [ces-d_1]; 2. I did not feel like eating; my appetite was poor [ces-d_2]; 3. I felt that I could not shake off the blues even with help from my family or friends [ces-d_3]; 4. I felt I was just as good as other people [ces-d_4]; 5. I had trouble keeping my mind on what I was doing [ces-d_5]; 6. I felt depressed [ces-d_6]; 7. I felt that everything I did was an effort [ces-d_7]; 8. I felt hopeful about the future [ces-d_8]; 9. I thought my life had been a failure [ces-d_9]; 10. I felt fearful [ces-d_10]; 11. My sleep was restless [ces-d_11]; 12. I was happy [ces-d_12]; 13. I talked less than usual [ces-d_13]; 14. I felt lonely [ces-d_14]; 15. People were unfriendly [ces-d_15]; 16. I enjoyed life [ces-d_16]; 17. I had crying spells [ces-d_17]; 18. I felt sad [ces-d_18]; 19. I felt that people dislike me [ces-d_19]; 20. I could not get “going” [ces-d_20].

- **Brief Symptom Inventory (BSI)** [5]

Depression: items 9-16-17-18-35-50

9. Thoughts of ending your life [bsi_9]

16. Feeling lonely [bsi_16]

17. Feeling blue [bsi_17]

18. Feeling no interest in things [bsi_18]

35. Feeling hopeless about the future [bsi_35]

50. Feelings of worthlessness [bsi_50]

- **Hospital Anxiety and Depression Scale (HADS)** [6]

Depression: items 2, 4, 6, 8, 10, 12 and 14.

2. I still enjoy the things I used to enjoy [hads_2]

4. I can laugh and see the funny side of things [hads_4]

6. I feel cheerful [hads_6]

8. I feel as if I am slowed down [hads_8]

10. I have lost interest in my appearance [hads_10]

12. I look forward with enjoyment to things [hads_12]

14. I can enjoy a good book or radio or TV program [hads_14]

- **Montgomery-Åsberg Depression Rating Scale (MADRS)** [7]

1. Apparent sadness (Despondency, gloom, and despair (more than just ordinary transient low spirits), reflected in speech, facial expression, and posture; rate by depth and inability to brighten up) [madrs_1]; 2. Reported sadness (Reports of depressed mood, regardless of whether it is reflected in appearance or not; includes low spirits, despondency, or the feeling of being beyond help and without hope) [madrs_2]; 3. Inner tension (Feelings of ill-defined discomfort, edginess, inner turmoil, mental tension mounting to either panic, dread, or anguish; rate by intensity, frequency, duration, and extent of reassurance called for) [madrs_3]; 4. Reduced sleep (Experience of reduced duration or depth of sleep compared to the patient's own normal pattern when well) [madrs_4]; 5. Reduced appetite (Feeling of loss of appetite; rate by loss of desire for food or the need to force oneself to eat) [madrs_5]; 6. Concentration difficulty (Difficulties in collecting one’s thoughts mounting to incapacitating lack of concentration; rate by intensity, frequency, and degree of incapacity produced) [madrs_6]; 7. Lassitude (Difficulty getting started or slowness initiating and performing everyday activities) [madrs_7]; 8. Inability to feel (Subjective experience of reduced interest in the surroundings or activities that normally give pleasure; the ability to react with adequate emotion to circumstances or people is reduced) [madrs_8]; 9. Pessimistic thoughts (Thoughts of guilt, inferiority, self-reproach, sinfulness, remorse, and ruin) [madrs_9]; 10. Suicidal thoughts (Feeling that life is not worth living, that a natural death would be welcome, suicidal thoughts, and the preparations for suicide; suicidal attempts should not in themselves influence the rating) [madrs_10].

- **Cornell Scale for Depression in Dementia (CSDD)** [8]

1. Mood-related signs
2. Anxiety (anxious expression, ruminations, worrying) [csdd_a1]
3. Sadness (sad expression, sad voice, tearfulness) [csdd_a2]
4. Lack of reactivity to pleasant events [csdd_a3]
5. Irritability (easily annoyed, short-tempered) [csdd_a4]
6. Behavioral disturbance
7. Agitation (restlessness, handwringing, hairpulling) [csdd_b1]
8. Retardation (slow movements, slow speech, slow reactions) [csdd_b2]
9. Multiple physical complaints (score 0 if GI symptoms only) [csdd_b3]
10. Loss of interest (less involved in usual activities) (score only if change occurred acutely, i.e., in less than 1 month) [csdd_b4]
11. Physical signs
12. Appetite loss (eating less than usual) [csdd_c1]
13. Weight loss (score 2 if greater than 5 Ib. in one month) [csdd_c2]
14. Lack of energy (fatigues easily, unable to sustain activities (score only if change occurred acutely, i.e., in less than 1 month) [csdd_c3]
15. Cyclic functions
16. Diurnal variation of mood (symptoms worse in the morning) [csdd_d1]
17. Difficulty falling asleep (later than usual for this individual) [csdd_d2]
18. Multiple awakenings during sleep [csdd_d3]
19. Early-morning awakening (earlier than usual for this individual) [csdd_d4]
20. Ideational disturbance
21. Suicide (feels like is not worth living, has suicidal wishes or makes suicide attempt) [csdd_e1]
22. Poor self-esteem (self-blame, self-depreciation, feelings of failure) [csdd_e2]
23. Pessimism (anticipation of the worst) [csdd_e3]
24. Mood-congruent delusions (delusions of poverty, illness or loss) [csdd_e4]

- **Neuropsychiatric Inventory (NPI)—Depression section** [9, 10]

Does the patient seem sad or depressed? Does he/she say that he/she feels sad or depressed? *(if yes, proceed to subquestions):* [npi_gen]

1. Does the patient have periods of tearfulness or sobbing that seem to indicate sadness? [npi_1]
2. Does the patient say or act as if he/she is sad or in low spirits? [npi_2]
3. Does the patient put him/herself down or say that he/she feels like a failure? [npi_3]
4. Does the patient say that he/she is a bad person or deserves to be punished? [npi_4]
5. Does the patient seem very discouraged or say that he/she has no future? [npi_5]
6. Does the patient say he/she is a burden to the family or that the family would be better off without him/her? [npi_6]
7. Does the patient express a wish for death or talk about killing him/herself? [npi_7]
8. Does the patient show any other signs of depression or sadness? [npi_8]

- **Depression Rating Scale (DRS) from the Resident Assessment Instrument (RAI)** [11, 12]

1. Made negative statements [drs_1]

2. Persistent anger with self or others [drs_2]

3. Expressions (including nonverbal) of what appear to be unrealistic fears [drs_3]

4. Repetitive health complaints [drs_4]

5. Repetitive anxious complaints/concerns (non-health-related) [drs_5]

6. Sad, pained, worried facial expression [drs_6]

7. Crying, tearfulness [drs_7]

**REFERENCES**

1. World Health Organization. ICD-11 for Mortality and Morbidity Statistics [Internet]. Geneva: World Health Organization; 2022; Available from: https://icd.who.int/browse/2024-01/mms/en.

2. American Psychiatric Association. Diagnostic and statistical manual of mental disorders (5th ed.). : American Psychiatric Publishing; 2013.

3. Yesavage JA, Brink TL, Rose TL, Lum O, Huang V, Adey M, et al. Development and validation of a geriatric depression screening scale: a preliminary report. J Psychiatr Res. 1982;17(1):37-49. PMID: 7183759. doi: 10.1016/0022-3956(82)90033-4.

4. Radloff LS. The CES-D Scale. Applied Psychological Measurement. 2016;1(3):385-401. doi: 10.1177/014662167700100306.

5. Derogatis LR. Brief Symptom Inventory (BSI): Administration, scoring, and procedures manual. 4th ed. Minneapolis, MN: National Computer Systems; 1993.

6. Zigmond AS, Snaith RP. The hospital anxiety and depression scale. Acta Psychiatr Scand. 1983 Jun;67(6):361-70. PMID: 6880820. doi: 10.1111/j.1600-0447.1983.tb09716.x.

7. Montgomery SA, Asberg M. A new depression scale designed to be sensitive to change. The British journal of psychiatry : the journal of mental science. 1979 Apr;134:382-9. PMID: 444788. doi: 10.1192/bjp.134.4.382.

8. Cornell Institute of Geriatric Psychiatry - Weill Medical College of Cornell University. The Cornell Scale for Depression in Dementia. 2002.

9. Cummings JL, Mega M, Gray K, Rosenberg-Thompson S, Carusi DA, Gornbein J. The Neuropsychiatric Inventory: comprehensive assessment of psychopathology in dementia. Neurology. 1994 Dec;44(12):2308-14. PMID: 7991117. doi: 10.1212/wnl.44.12.2308.

10. Kaufer DI, Cummings JL, Christine D, Bray T, Castellon S, Masterman D, et al. Assessing the impact of neuropsychiatric symptoms in Alzheimer's disease: the Neuropsychiatric Inventory Caregiver Distress Scale. J Am Geriatr Soc. 1998 Feb;46(2):210-5. PMID: 9475452. doi: 10.1111/j.1532-5415.1998.tb02542.x.

11. interRAI. Instruments overview [Internet]. Available from: https://www.interrai.org/instruments/.

12. Burrows AB, Morris JN, Simon SE, Hirdes JP, Phillips C. Development of a minimum data set-based depression rating scale for use in nursing homes. Age Ageing. 2000 Mar;29(2):165-72. PMID: 10791452. doi: 10.1093/ageing/29.2.165.
