# Supplementary figures and images for: Building Consensus on the Relevant Criteria to Screen for Depressive Symptoms Among Near-Centenarians and Centenarians: Modified e-Delphi Study
Source: JMIR Aging. 2025 Mar 5;8:e64352. doi: 10.2196/64352 (PMC11923476; doi:10.2196/64352)

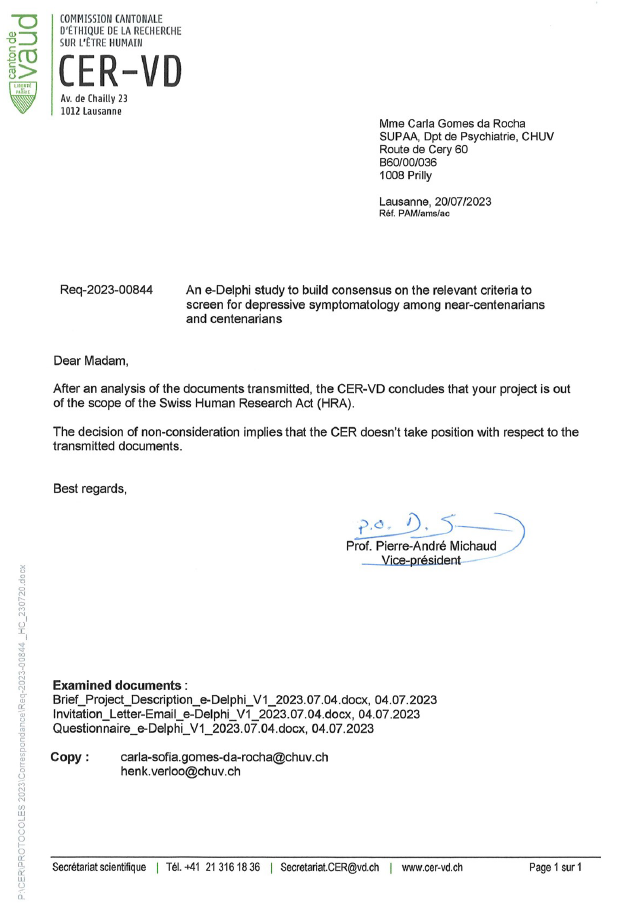

Supplement: Multimedia Appendix 2 [file aging_v8i1e64352_app2.docx]
