# Supplementary material for: Building Consensus on the Relevant Criteria to Screen for Depressive Symptoms Among Near-Centenarians and Centenarians: Modified e-Delphi Study
Source: JMIR Aging. 2025 Mar 5;8:e64352. doi: 10.2196/64352 (PMC11923476; doi:10.2196/64352)
Supplement: Multimedia Appendix 3 [file aging_v8i1e64352_app3.docx]

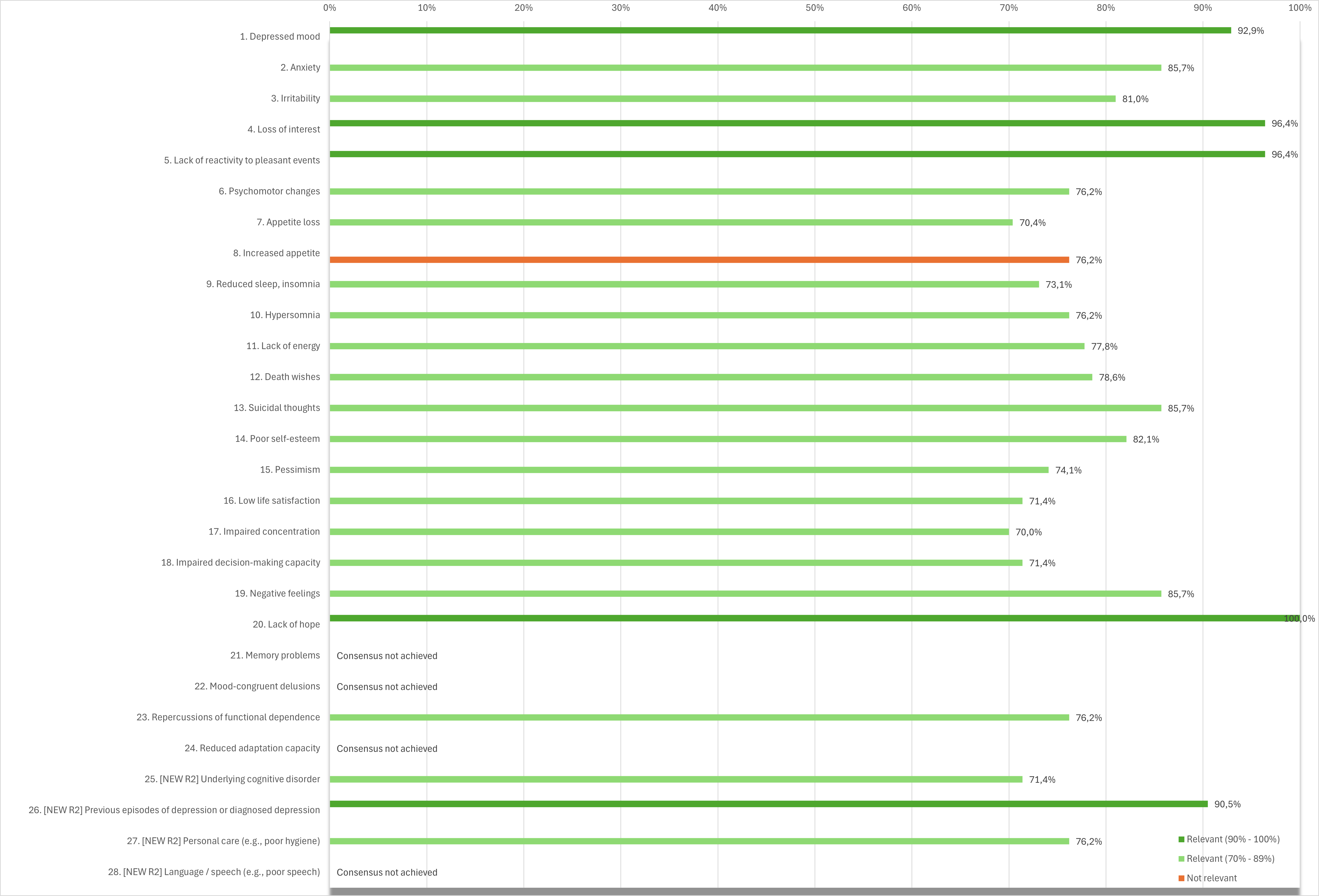


Figure S1: Graphical overview of the consensus rates for the potentially relevant dimensions for depression screening in near-centenarians and centenarians.


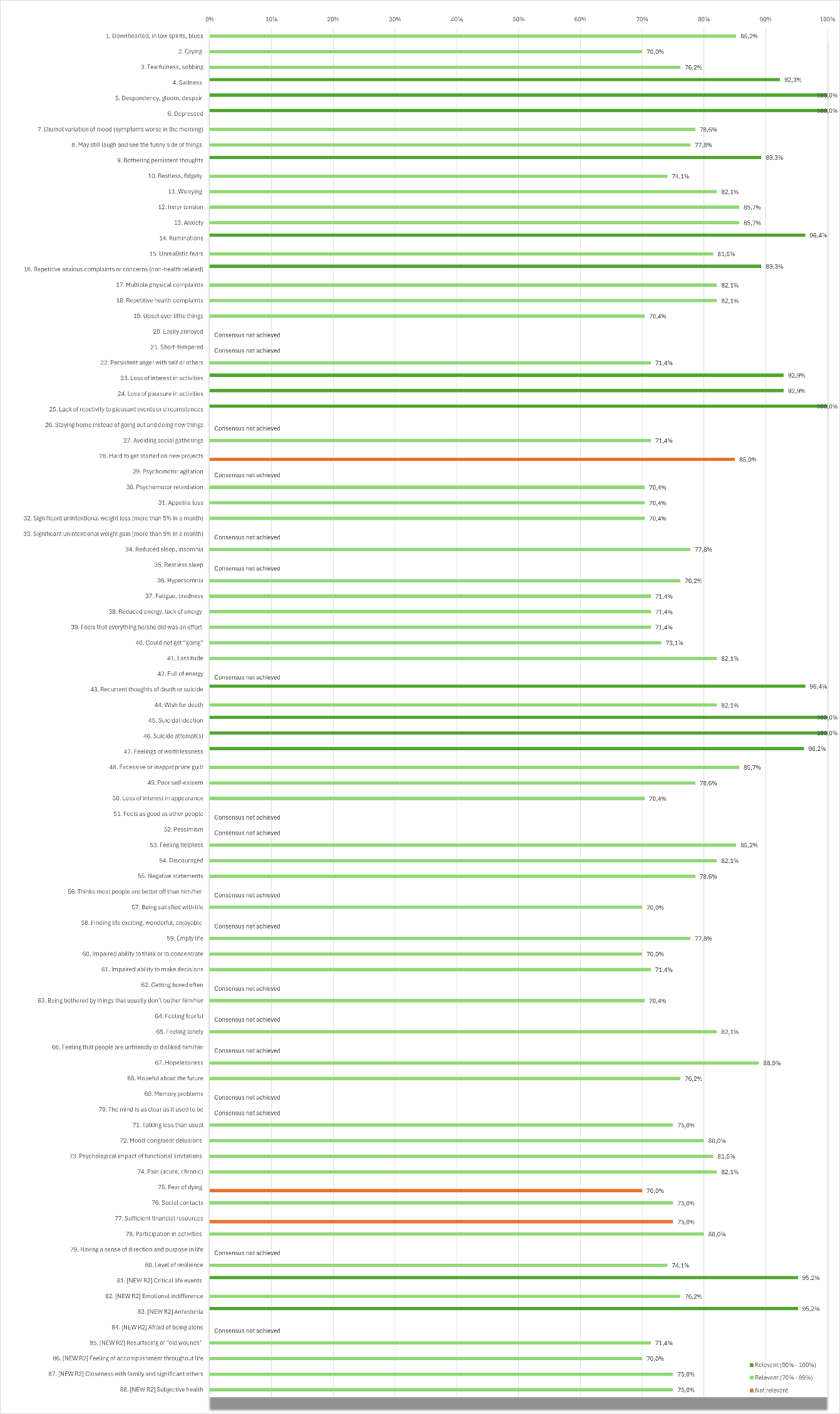


Figure S2: Graphical overview of the consensus rates for the potentially relevant criteria for depression screening in near-centenarians and centenarians.


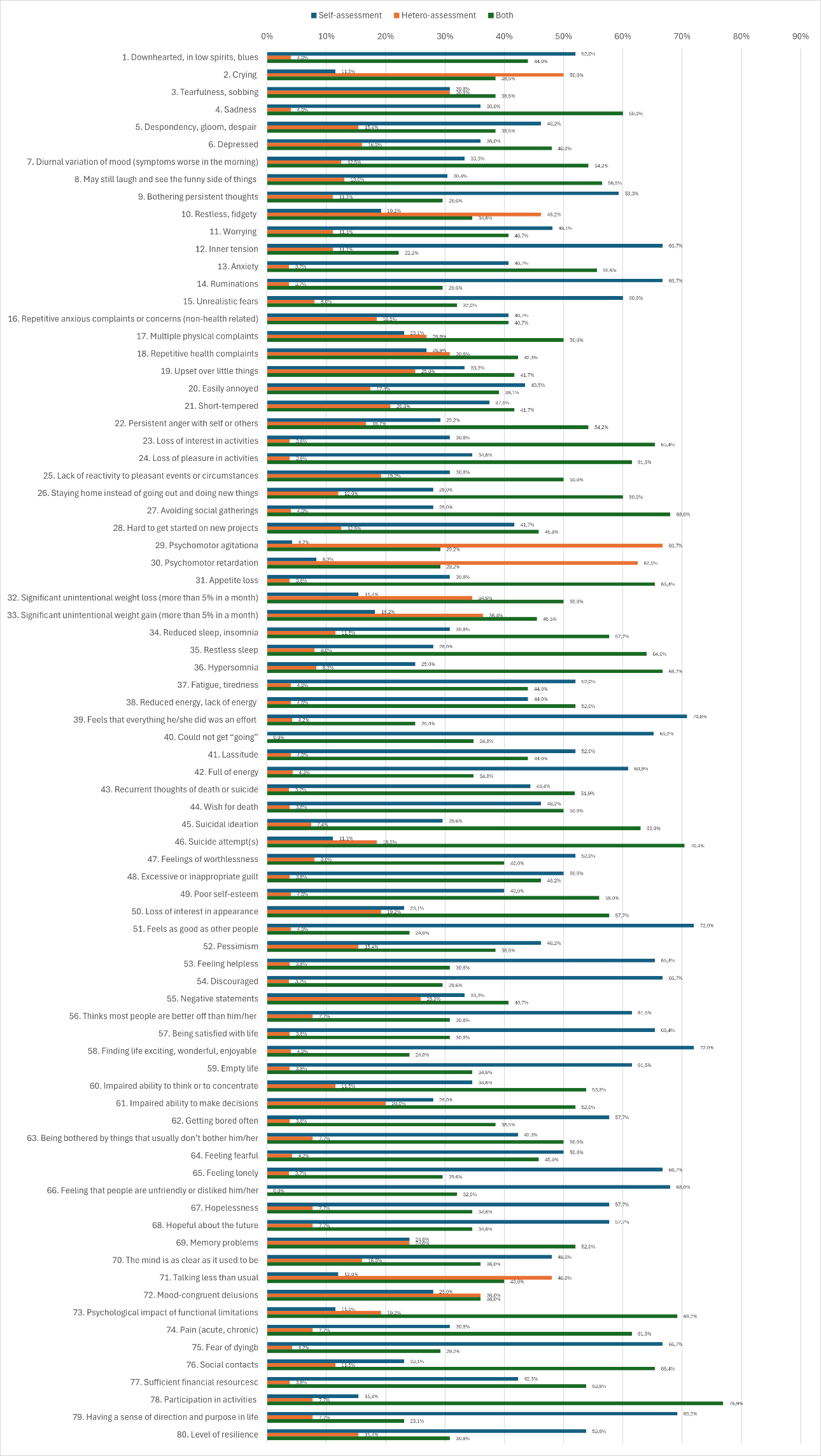


Figure S3: Graphical overview of the consensus rates for types of assessment methods for the potentially relevant criteria for depression screening in near-centenarians and centenarians.
