# Supplementary material for: Building Consensus on the Relevant Criteria to Screen for Depressive Symptoms Among Near-Centenarians and Centenarians: Modified e-Delphi Study
Source: JMIR Aging. 2025 Mar 5;8:e64352. doi: 10.2196/64352 (PMC11923476; doi:10.2196/64352)
Supplement: Multimedia Appendix 4 [file aging_v8i1e64352_app4.docx]

Consensus level on potential criteria for depression screening.^a^

|  | Round 1 (n=28) | | | Round 2 (n=21) | | | Round 3 (n=20) | | |
| --- | --- | --- | --- | --- | --- | --- | --- | --- | --- |
|  | Level of consensus at ≥70% | Result | Participants, n (%) | Level of consensus at ≥70% | Result | Participants, n (%) | Level of consensus at ≥70% | Result | Participants, n (%) |
|  | | | | | | | | | |
| Downhearted, in low spirits, or blues | Achieved | Relevant | 23 (85)^b^ | —^c^ | — | — | — | — | — |
| Crying | Not achieved | N/A^d^ | N/A | Not achieved | N/A | N/A | Achieved | Relevant | 14 (70)^e^ |
| Tearfulness and sobbing | Not achieved | N/A | N/A | Achieved | Relevant | 16 (76)^f^ | — | — | — |
| Sadness | Achieved | Relevant | 24 (92)^g^ | — | — | — | — | — | — |
| Despondency, gloom, and despair | Achieved | Relevant | 25 (100)^h^ | — | — | — | — | — | — |
| Depressed | Achieved | Relevant | 27 (100)^b^ | — | — | — | — | — | — |
| Diurnal variation of mood (symptoms worse in the morning) | Achieved | Relevant | 22 (79)^i^ | — | — | — | — | — | — |
| May still laugh and see the funny side of things | Achieved | Relevant | 21 (78)^b^ | — | — | — | — | — | — |
| Bothering persistent thoughts | Achieved | Relevant | 25 (89)^i^ | — | — | — | — | — | — |
| Restless and fidgety | Achieved | Relevant | 20 (74)^b^ | — | — | — | — | — | — |
| Worrying | Achieved | Relevant | 23 (82)^i^ | — | — | — | — | — | — |
| Inner tensión | Achieved | Relevant | 24 (86)^i^ | — | — | — | — | — | — |
| Anxiety | Achieved | Relevant | 24 (86)^i^ | — | — | — | — | — | — |
| Ruminations | Achieved | Relevant | 27 (96)^i^ | — | — | — | — | — | — |
| Unrealistic fears | Achieved | Relevant | 22 (81)^b^ | — | — | — | — | — | — |
| Repetitive anxious complaints or concerns (non–health-related) | Achieved | Relevant | 25 (89)^i^ | — | — | — | — | — | — |
| Multiple physical complaints | Achieved | Relevant | 23 (82)^i^ | — | — | — | — | — | — |
| Repetitive health complaints | Achieved | Relevant | 23 (82)^i^ | — | — | — | — | — | — |
| Upset over little things | Achieved | Relevant | 19 (70)^b^ | — | — | — | — | — | — |
| Easily annoyed | Not achieved | N/A | N/A | Not achieved | N/A | N/A | Not achieved | N/A | N/A |
| Short-tempered | Not achieved | N/A | N/A | Not achieved | N/A | N/A | Not achieved | N/A | N/A |
| Persistent anger with self or others | Achieved | Relevant | 20 (71)^i^ | — | — | — | — | — | — |
| Loss of interest in activities | Achieved | Relevant | 26 (93)^i^ | — | — | — | — | — | — |
| Loss of pleasure in activities | Achieved | Relevant | 26 (93)^i^ | — | — | — | — | — | — |
| Lack of reactivity to pleasant events or circumstances | Achieved | Relevant | 28 (100)^i^ | — | — | — | — | — | — |
| Staying home instead of going out and doing new things | Not achieved | N/A | N/A | Not achieved | N/A | N/A | Not achieved | N/A | N/A |
| Avoiding social gatherings | Achieved | Relevant | 20 (71)^i^ | — | — | — | — | — | — |
| Hard to get started on new projects | Not achieved | N/A | N/A | Not achieved | N/A | N/A | Achieved | Not relevant | 17 (85)^e^ |
| Psychomotor agitation | Not achieved | N/A | N/A | Not achieved | N/A | N/A | Not achieved | N/A | N/A |
| Psychomotor retardation | Achieved | Relevant | 19 (70)^b^ | — | — | — | — | — | — |
| Appetite los | Achieved | Relevant | 19 (70)^b^ | — | — | — | — | — | — |
| Significant unintentional weight loss (more than 5% in a month) | Achieved | Relevant | 19 (70)^b^ | — | — | — | — | — | — |
| Significant unintentional weight gain (more than 5% in a month) | Not achieved | N/A | N/A | Not achieved | N/A | N/A | Not achieved | N/A | N/A |
| Reduced sleep or insomnia | Achieved | Relevant | 21 (78)^b^ | — | — | — | — | — | — |
| Restless sleep | Not achieved | N/A | N/A | Not achieved | N/A | N/A | Not achieved | N/A | N/A |
| Hypersomnia | Not achieved | N/A | N/A | Achieved | Relevant | 16 (76)^f^ | — | — | — |
| Fatigue and tiredness | Not achieved | N/A | N/A | Achieved | Relevant | 15 (71)^f^ | — | — | — |
| Reduced energy or lack of energy | Achieved | Relevant | 20 (71)^i^ | — | — | — | — | — | — |
| Feels that everything he/she does is an effort | Not achieved | N/A | N/A | Achieved | Relevant | 15 (71)^f^ | — | — | — |
| Could not get “going” | Achieved | Relevant | 19 (73)^g^ | — | — | — | — | — | — |
| Lassitude | Achieved | Relevant | 23 (82)^i^ | — | — | — | — | — | — |
| Full of energy | Not achieved | N/A | N/A | Not achieved | N/A | N/A | Not achieved | N/A | N/A |
| Recurrent thoughts of death or suicide | Achieved | Relevant | 27 (96)^i^ | — | — | — | — | — | — |
| Wish for death | Achieved | Relevant | 23 (82)^i^ | — | — | — | — | — | — |
| Suicidal ideation | Achieved | Relevant | 28 (100)^i^ | — | — | — | — | — | — |
| Suicide attempts | Achieved | Relevant | 28 (100)^i^ | — | — | — | — | — | — |
| Feelings of worthlessness | Achieved | Relevant | 25 (96)^g^ | — | — | — | — | — | — |
| Excessive or inappropriate guilt | Achieved | Relevant | 24 (86)^i^ | — | — | — | — | — | — |
| Poor self-esteem | Achieved | Relevant | 22 (79)^i^ | — | — | — | — | — | — |
| Loss of interest in appearance | Achieved | Relevant | 19 (70)^b^ | — | — | — | — | — | — |
| Feels as good as other people | Not achieved | N/A | N/A | Not achieved | N/A | N/A | Not achieved | N/A | N/A |
| Pessimism | Not achieved | N/A | N/A | Not achieved | N/A | N/A | Not achieved | N/A | N/A |
| Feeling helpless | Achieved | Relevant | 23 (85)^b^ | — | — | — | — | — | — |
| Discouraged | Achieved | Relevant | 23 (82)^i^ | — | — | — | — | — | — |
| Negative statements | Achieved | Relevant | 22 (79)^i^ | — | — | — | — | — | — |
| Thinks most people are better off than him/her | Not achieved | N/A | N/A | Not achieved | N/A | N/A | Not achieved | N/A | N/A |
| Being satisfied with life | Not achieved | N/A | N/A | Not achieved | N/A | N/A | Achieved | Relevant | 14 (70)^e^ |
| Finding life exciting, wonderful, and enjoyable | Not achieved | N/A | N/A | Not achieved | N/A | N/A | Not achieved | N/A | N/A |
| Empty life | Achieved | Relevant | 21 (78)^b^ | — | — | — | — | — | — |
| Impaired ability to think or to concéntrate | Not achieved | N/A | N/A | Not achieved | N/A | N/A | Achieved | Relevant | 14 (70)^e^ |
| Impaired ability to make decisions | Not achieved | N/A | N/A | Achieved | Relevant | 15 (71)^f^ | — | — | — |
| Getting bored often | Not achieved | N/A | N/A | Not achieved | N/A | N/A | Not achieved | N/A | N/A |
| Being bothered by things that usually don’t bother him/her | Achieved | Relevant | 19 (70)^b^ | — | — | — | — | — | — |
| Feeling fearful | Not achieved | N/A | N/A | Not achieved | N/A | N/A | Not achieved | N/A | N/A |
| Feeling lonely | Achieved | Relevant | 23 (82)^i^ | — | — | — | — | — | — |
| Feeling that people are unfriendly or dislike him/her | Not achieved | N/A | N/A | Not achieved | N/A | N/A | Not achieved | N/A | N/A |
| Hopelessness | Achieved | Relevant | 24 (89)^b^ | — | — | — | — | — | — |
| Hopeful about the future | Not achieved | N/A | N/A | Achieved | Relevant | 16 (76)^f^ | — | — | — |
| Memory problems | Not achieved | N/A | N/A | Not achieved | N/A | N/A | Not achieved | N/A | N/A |
| The mind is as clear as it used to be | Not achieved | N/A | N/A | Not achieved | N/A | N/A | Not achieved | N/A | N/A |
| Talking less than usual | Achieved | Relevant | 21 (75)^i^ | — | — | — | — | — | — |
| Mood-congruent delusions (*delusions of poverty, illness, or loss*) | Not achieved | N/A | N/A | Achieved | Relevant | 16 (80)^e^ | — | — | — |
| Psychological impact of functional limitations (*incapacity to perform activities of daily living independently*) | Achieved | Relevant | 22 (81)^b^ | — | — | — | — | — | — |
| Pain (acute or chronic) | Achieved | Relevant | 23 (82)^i^ | — | — | — | — | — | — |
| Fear of dying | Not achieved | N/A | N/A | Not achieved | N/A | N/A | Achieved | Not relevant | 14 (70)^e^ |
| Social contacts | Achieved | Relevant | 21 (75)^i^ | — | — | — | — | — | — |
| Sufficient financial resources | Not achieved | N/A | N/A | Not achieved | N/A | N/A | Achieved | Not relevant | 15 (75)^e^ |
| Participation in activities (*eg, reading; using the computer or smartphone, television, or radio; playing an instrument or games; physical activity; gardening; going to the cinema, the theater, a café, or a restaurant; attending religious services; visiting people; or associative activities*) | Not achieved | N/A | N/A | Not achieved | N/A | N/A | Achieved | Relevant | 16 (80)^e^ |
| Having a sense of direction and purpose in life | Not achieved | N/A | N/A | Not achieved | N/A | N/A | Not achieved | N/A | N/A |
| Level of resilience | Achieved | Relevant | 20 (74)^b^ | — | — | — | — | — | — |
| Critical life events (eg, loss of a child or another loved one; added in round 2) | N/A | N/A | N/A | Achieved | Relevant | 20 (95)^f^ | — | — | — |
| Emotional indifference (added in round 2) | N/A | N/A | N/A | Achieved | Relevant | 16 (76)^f^ | — | — | — |
| Anhedonia (added in round 2) | N/A | N/A | N/A | Achieved | Relevant | 20 (95)^f^ | — | — | — |
| Afraid of being alone (added in round 2) | N/A | N/A | N/A | Not achieved | N/A | N/A | Not achieved | N/A | N/A |
| Resurfacing of “old wounds” (eg, long-standing conflicts with children; added in round 2) | N/A | N/A | N/A | Achieved | Relevant | 15 (71)^f^ | — | — | — |
| Feeling of accomplishment throughout life (added in round 2) | N/A | N/A | N/A | Not achieved | N/A | N/A | Achieved | Relevant | 14 (70)^e^ |
| Closeness with family and significant others (added in round 2) | N/A | N/A | N/A | Not achieved | N/A | N/A | Achieved | Relevant | 15 (75)^e^ |
| Subjective health (added in round 2) | N/A | N/A | N/A | Achieved | Relevant | 15 (75)^e^ | — | — | — |

^a^This list includes 8 criteria suggested by the expert participants in round 1 and incorporated into the subsequent rounds: *critical life events (eg, loss of a child or another loved one)*, *emotional indifference*, *anhedonia*, *afraid of being alone*, *resurfacing of “old wounds” (eg, long-standing conflicts with children)*, *feeling of accomplishment throughout life*, *closeness with family and significant others*, and *subjective health*.

^b^n=27.

^c^Data are not available for the corresponding entries.

^d^N/A: not applicable.

^e^n=20.

^f^n=21.

^g^n=26.

^h^n=25.

^i^n=28.
