# Supplementary material for: Building Consensus on the Relevant Criteria to Screen for Depressive Symptoms Among Near-Centenarians and Centenarians: Modified e-Delphi Study
Source: JMIR Aging. 2025 Mar 5;8:e64352. doi: 10.2196/64352 (PMC11923476; doi:10.2196/64352)
Supplement: Multimedia Appendix 5 [file aging_v8i1e64352_app5.docx]

A. Do you consider the duration of signs or symptoms to be important? (N = 28)

- Yes: 28 (100%)

A.1 [*If yes*] In your opinion, what is the minimum duration to consider the signs or symptoms as clinically significant?

|  | R1 (N = 25)  n (%) |
| --- | --- |
| 1 to 3 days | 2 (8.0) |
| 1 week | 3 (12.0) |
| 2 weeks | 9 (36.0) |
| 3 weeks | 3 (12.0) |
| 4 weeks | 4 (16.0) |
| 7 weeks | 0 (0.0) |
| 8 weeks | 2 (8.0) |
| 6 months | 2 (8.0) |

B. Do you believe that the minimum duration for signs/symptoms to be considered clinically significant differs between a recurrence in someone with chronic depression and a new onset of depression in this age group? (N = 21)

- Yes: 13 (61.9%)
- No: 8 (38.1%)

B.1 [*If yes*] For a recurrence in someone with chronic depression, what do you believe is the minimum duration for the signs/symptoms to be considered clinically significant? (In days, weeks or months)

|  | R2 (N = 13)  n (%) |
| --- | --- |
| 1 week | 3 (23.1) |
| 2 weeks | 9 (69.2) |
| 7 weeks | 1 (7.7) |

B.2 [*If yes*] For a new onset of depression, what do you believe is the minimum duration for the signs/symptoms to be considered clinically significant? (In days, weeks or months)

|  | R2 (N = 13)  n (%) |
| --- | --- |
| 3 days | 1 (7.7) |
| 1 week | 2 (15.4) |
| 2 weeks | 2 (15.4) |
| 3 weeks | 2 (15.4) |
| 4 weeks | 5 (38.5) |
| 9 weeks | 1 (7.7) |

C. Do you believe the number of signs/symptoms is important to consider when assessing potential depression in this age group? (N = 21)

- Yes: 13 (61.9%)
- No: 6 (28.6%)
- I don’t know: 2 (9.5%)

C.1 [*If yes*] In your opinion, what is the minimum number of signs/symptoms that should warrant clinical concern for possible depression in this age group?

| Min. number of signs/symptoms | R2 (N = 12)  n (%) |
| --- | --- |
| 1 | 1 (8.3) |
| 2 | 4 (33.3) |
| 3 | 4 (33.3) |
| 4 | 1 (8.3) |
| 5 | 2 (16.7) |

D. Do you believe the frequency of signs/symptoms is an important factor to consider when assessing potential depression in this age group? (N = 20)

- Yes: 20 (100%)

D.1 [*If yes*] In your opinion, at what frequency should the presence of signs/symptoms raise clinical concern for potential depression (e.g., continuously, several times per day, several times per week ...)?

|  | R2 (N = 19)  n (%) |
| --- | --- |
| Continuously | 1 (5.3) |
| Several times per day | 9 (47.4) |
| Near daily | 3 (15.8) |
| Several times per week | 6 (31.6) |

E. Do you believe the severity of signs/symptoms is an important factor to consider when assessing potential depression in this age group? (N = 20)

- Yes: 18 (90.0%)
- No: 2 (10.0%)

E.1 [*If yes*] At what level of severity (on a scale of 1-10, with 10 being the most severe) would signs/symptoms warrant clinical concern for potential depression in this age group? (**N = 18)**

| Mean (SD) | 4.9 (1.4) |
| --- | --- |
| Median (IQR) | 5.0 (2.0) |
| Level of severity | n (%) |
| 2 | 1 (5.6) |
| 3 | 2 (11.1) |
| 4 | 4 (22.2) |
| 5 | 4 (22.2) |
| 6 | 5 (27.8) |
| 7 | 2 (11.1) |
