# Supplementary material for: Building Consensus on the Relevant Criteria to Screen for Depressive Symptoms Among Near-Centenarians and Centenarians: Modified e-Delphi Study
Source: JMIR Aging. 2025 Mar 5;8:e64352. doi: 10.2196/64352 (PMC11923476; doi:10.2196/64352)
Supplement: Multimedia Appendix 6 [file aging_v8i1e64352_app6.docx]

### **Steering committee’s decision on dimensions lacking consensus after three e-Delphi rounds**

The committee's evaluations led to the following determinations:

- Dimension *Memory problems* (dim. 21) and dimension *Language / speech (e.g., poor speech*; dim. 28): these dimensions were classified as ‘not relevant’ for inclusion in the depression screening for individuals aged 95 and older. The committee felt that these dimensions may not capture the critical elements of depressive symptoms in this specific age group because they may overlap with cognitive deficits that are prevalent in older adults but not necessarily indicative of depression.
- Dimension *Mood-congruent delusions* (dim. 22) and dimension *Reduced adaptation capacity* (dim. 24): these dimensions were classified as ‘relevant’ for inclusion in the depression screening for individuals aged 95 and older. The committee concluded that mood-congruent delusions may be particularly indicative of depressive severity, while adaptation capacity is crucial for assessing an individual’s capacity to manage daily stress and adapt to changes, factors that can significantly affect their overall mental health.

### **Steering committee’s decision on criteria lacking consensus after three e-Delphi rounds**

The committee's evaluations led to the following determinations:

- The following criteria were classified as ‘not relevant’ for inclusion in depression screening for individuals aged 95 and older: *Easily annoyed* (crit. 20), *Staying home instead of going out and doing new things* (crit. 26), *Psychomotor agitation* (crit. 29), *Significant unintentional weight gain* (more than 5% in a month) (crit. 33), *Full of energy* (crit. 42), *Feels as good as other people* (crit. 51), *Thinks most people are better off than him/her* (crit. 56), *Finding life exciting, wonderful, enjoyable* (crit. 58), *Getting bored often* (crit. 62), *Feeling that people are unfriendly or disliked him/her* (crit. 66), *Memory problems* (crit. 69), and *The mind is as clear as it used to be* (crit. 70).

Regarding criteria 20, 26, 33, 42, 51, 56, 58, 62, and 66, the committee concluded that they may not significantly reflect depressive symptoms in this age group, possibly because they could be more indicative of personality traits or lifestyle choices rather than underlying depressive disorders. For criterion 29, the committee determined that it may often result from other medical conditions (e.g., Alzheimer’s disease, delirium, anxiety disorders), making it less specific as an indicator of depression in the very old. Additionally, criteria 69 and 70 were excluded in line with the dimension *Memory problems* (dim. 21) being deemed ‘not relevant’, as previously presented. These memory-related issues may often reflect age-related cognitive decline or dementia rather than depressive disorders, thus being less specific indicators of depression in individuals aged 95 and older.

- The following criteria were classified as ‘relevant’ for inclusion in depression screening for individuals aged 95 and older: *Short-tempered* (crit. 21), *Restless sleep* (crit. 35), *Pessimism* (crit. 52), *Feeling fearful* (crit. 64), *Having a sense of direction and purpose in life* (crit. 79), and *Afraid of being alone* (crit. 84). The committee concluded that these criteria could be critical in identifying depressive conditions as they directly relate to emotional regulation, sleep disturbances, fear responses, negative outlooks, existential engagement, and social dependency, which can be seen as particularly important indicators of mental health challenges in older adults.
