# Supplementary material for: Building Consensus on the Relevant Criteria to Screen for Depressive Symptoms Among Near-Centenarians and Centenarians: Modified e-Delphi Study
Source: JMIR Aging. 2025 Mar 5;8:e64352. doi: 10.2196/64352 (PMC11923476; doi:10.2196/64352)
Supplement: Multimedia Appendix 7 [file aging_v8i1e64352_app7.docx]

Findings on Preferred Assessment Methods for Depression Screening Criteria

|  | Round 1  N = 28 | | |
| --- | --- | --- | --- |
|  | Self-assessment  n/N (%) | Hetero-assessment  n/N (%) | Both  n/N (%) |
|  |  |  |  |
| 1. **Downhearted, in low spirits, blues** | **13/25 (52.0)** | 1/25 (4.0) | 11/25 (44.0) |
| 1. **Crying** | 3/26 (11.5) | **13/26 (50.0)** | 10/26 (38.5) |
| 1. **Tearfulness, sobbing** | 8/26 (30.8) | 8/26 (30.8) | 10/26 (38.5) |
| 1. **Sadness** | 9/25 (36.0) | 1/25 (4.0) | **15/25 (60.0)** |
| 1. **Despondency, gloom, despair** | 12/26 (46.2) | 4/26 (15.4) | 10/26 (38.5) |
| 1. **Depressed** | 9/25 (36.0) | 4/25 (16.0) | 12/25 (48.0) |
| 1. **Diurnal variation of mood (symptoms worse in the morning)** | 8/24 (33.3) | 3/24 (12.5) | **13/24 (54.2)** |
| 1. **May still laugh and see the funny side of things** | 7/23 (30.4) | 3/23 (13.0) | **13/23 (56.5)** |
| 1. **Bothering persistent thoughts** | **16/27 (59.3)** | 3/27 (11.1) | 8/27 (29.6) |
| 1. **Restless, fidgety** | 5/26 (19.2) | 12/26 (46.2) | 9/26 (34.6) |
| 1. **Worrying** | 13/27 (48.1) | 3/27 (11.1) | 11/27 (40.7) |
| 1. **Inner tension** | **18/27 (66.7)** | 3/27 (11.1) | 6/27 (22.2) |
| 1. **Anxiety** | 11/27 (40.7) | 1/27 (3.7) | **15/27 (55.6)** |
| 1. **Ruminations** | **18/27 (66.7)** | 1/27 (3.7) | 8/27 (29.6) |
| 1. **Unrealistic fears** | **15/25 (60.0)** | 2/25 (8.0) | 8/25 (32.0) |
| 1. **Repetitive anxious complaints or concerns (non-health related)** | 11/27 (40.7) | 5/27 (18.5) | 11/27 (40.7) |
| 1. **Multiple physical complaints** | 6/26 (23.1) | 7/26 (26.9) | **13/26 (50.0)** |
| 1. **Repetitive health complaints** | 7/26 (26.9) | 8/26 (30.8) | 11/26 (42.3) |
| 1. **Upset over little things** | 8/24 (33.3) | 6/24 (25.0) | 10/24 (41.7) |
| 1. **Easily annoyed** | 10/23 (43.5) | 4/23 (17.4) | 9/23 (39.1) |
| 1. **Short-tempered** | 9/24 (37.5) | 5/24 (20.8) | 10/24 (41.7) |
| 1. **Persistent anger with self or others** | 7/24 (29.2) | 4/24 (16.7) | **13/24 (54.2)** |
| 1. **Loss of interest in activities** | 8/26 (30.8) | 1/26 (3.8) | **17/26 (65.4)** |
| 1. **Loss of pleasure in activities** | 9/26 (34.6) | 1/26 (3.8) | **16/26 (61.5)** |
| 1. **Lack of reactivity to pleasant events or circumstances** | 8/26 (30.8) | 5/26 (19.2) | **13/26 (50.0)** |
| 1. **Staying home instead of going out and doing new things** | 7/25 (28.0) | 3/25 (12.0) | **15/25 (60.0)** |
| 1. **Avoiding social gatherings** | 7/25 (28.0) | 1/25 (4.0) | **17/25 (68.0)** |
| 1. **Hard to get started on new projects** | 10/24 (41.7) | 3/24 (12.5) | 11/24 (45.8) |
| 1. **Psychomotor agitation ^a^** | 1/24 (4.2) | **16/24 (66.7)** | 7/24 (29.2) |
| 1. **Psychomotor retardation** | 2/24 (8.3) | **15/24 (62.5)** | 7/24 (29.2) |
| 1. **Appetite loss** | 8/26 (30.8) | 1/26 (3.8) | **17/26 (65.4)** |
| 1. **Significant unintentional weight loss (more than 5% in a month)** | 4/26 (15.4) | 9/26 (34.6) | **13/26 (50.0)** |
| 1. **Significant unintentional weight gain (more than 5% in a month)** | 4/22 (18.2) | 8/22 (36.4) | 10/22 (45.5) |
| 1. **Reduced sleep, insomnia** | 8/26 (30.8) | 3/26 (11.5) | **15/26 (57.7)** |
| 1. **Restless sleep** | 7/25 (28.0) | 2/25 (8.0) | **16/25 (64.0)** |
| 1. **Hypersomnia** | 6/24 (25.0) | 2/24 (8.3) | **16/24 (66.7)** |
| 1. **Fatigue, tiredness** | **13/25 (52.0)** | 1/25 (4.0) | 11/25 (44.0) |
| 1. **Reduced energy, lack of energy** | 11/25 (44.0) | 1/25 (4.0) | **13/25 (52.0)** |
| 1. **Feels that everything he/she did was an effort** | **17/24 (70.8)** | 1/24 (4.2) | 6/24 (25.0) |
| 1. **Could not get “going”** | **15/23 (65.2)** | 0/23 (0.0) | 8/23 (34.8) |
| 1. **Lassitude** | **13/25 (52.0)** | 1/25 (4.0) | 11/25 (44.0) |
| 1. **Full of energy** | **14/23 (60.9)** | 1/23 (4.3) | 8/23 (34.8) |
| 1. **Recurrent thoughts of death or suicide** | 12/27 (44.4) | 1/27 (3.7) | **14/27 (51.9)** |
| 1. **Wish for death** | 12/26 (46.2) | 1/26 (3.8) | **13/26 (50.0)** |
| 1. **Suicidal ideation** | 8/27 (29.6) | 2/27 (7.4) | **17/27 (63.0)** |
| 1. **Suicide attempt(s)** | 3/27 (11.1) | 5/27 (18.5) | **19/27 (70.4)** |
| 1. **Feelings of worthlessness** | **13/25 (52.0)** | 2/25 (8.0) | 10/25 (40.0) |
| 1. **Excessive or inappropriate guilt** | **13/26 (50.0)** | 1/26 (3.8) | 12/26 (46.2) |
| 1. **Poor self-esteem** | 10/25 (40.0) | 1/25 (4.0) | **14/25 (56.0)** |
| 1. **Loss of interest in appearance** | 6/26 (23.1) | 5/26 (19.2) | **15/26 (57.7)** |
| 1. **Feels as good as other people** | **18/25 (72.0)** | 1/25 (4.0) | 6/25 (24.0) |
| 1. **Pessimism** | 12/26 (46.2) | 4/26 (15.4) | 10/26 (38.5) |
| 1. **Feeling helpless** | **17/26 (65.4)** | 1/26 (3.8) | 8/26 (30.8) |
| 1. **Discouraged** | **18/27 (66.7)** | 1/27 (3.7) | 8/27 (29.6) |
| 1. **Negative statements** | 9/27 (33.3) | 7/27 (25.9) | 11/27 (40.7) |
| 1. **Thinks most people are better off than him/her** | **16/26 (61.5)** | 2/26 (7.7) | 8/26 (30.8) |
| 1. **Being satisfied with life** | **17/26 (65.4)** | 1/26 (3.8) | 8/26 (30.8) |
| 1. **Finding life exciting, wonderful, enjoyable** | **18/25 (72.0)** | 1/25 (4.0) | 6/25 (24.0) |
| 1. **Empty life** | **16/26 (61.5)** | 1/26 (3.8) | 9/26 (34.6) |
| 1. **Impaired ability to think or to concentrate** | 9/26 (34.6) | 3/26 (11.5) | **14/26 (53.8)** |
| 1. **Impaired ability to make decisions** | 7/25 (28.0) | 5/25 (20.0) | **13/25 (52.0)** |
| 1. **Getting bored often** | **15/26 (57.7)** | 1/26 (3.8) | 10/26 (38.5) |
| 1. **Being bothered by things that usually don’t bother him/her** | 11/26 (42.3) | 2/26 (7.7) | **13/26 (50.0)** |
| 1. **Feeling fearful** | **12/24 (50.0)** | 1/24 (4.2) | 11/24 (45.8) |
| 1. **Feeling lonely** | **18/27 (66.7)** | 1/27 (3.7) | 8/27 (29.6) |
| 1. **Feeling that people are unfriendly or disliked him/her** | **17/25 (68.0)** | 0/25 (0.0) | 8/25 (32.0) |
| 1. **Hopelessness** | **15/26 (57.7)** | 2/26 (7.7) | 9/26 (34.6) |
| 1. **Hopeful about the future** | **15/26 (57.7)** | 2/26 (7.7) | 9/26 (34.6) |
| 1. **Memory problems** | 6/25 (24.0) | 6/25 (24.0) | **13/25 (52.0)** |
| 1. **The mind is as clear as it used to be** | 12/25 (48.0) | 4/25 (16.0) | 9/25 (36.0) |
| 1. **Talking less than usual** | 3/25 (12.0) | 12/25 (48.0) | 10/25 (40.0) |
| 1. **Mood-congruent delusions (delusions of poverty, illness or loss)** | 7/25 (28.0) | 9/25 (36.0) | 9/25 (36.0) |
| 1. **Psychological impact of functional limitations (incapacity to perform activities of daily living independently)** | 3/26 (11.5) | 5/26 (19.2) | **18/26 (69.2)** |
| 1. **Pain (acute, chronic)** | 8/26 (30.8) | 2/26 (7.7) | **16/26 (61.5)** |
| 1. **Fear of dying ^a^** | **16/24 (66.7)** | 1/24 (4.2) | 7/24 (29.2) |
| 1. **Social contacts** | 6/26 (23.1) | 3/26 (11.5) | **17/26 (65.4)** |
| 1. **Sufficient financial resources ^a^** | 11/26 (42.3) | 1/26 (3.8) | **14/26 (53.8)** |
| 1. **Participation in activities (reading, using the computer/smartphone, TV/radio, playing an instrument/games, physical activity, gardening, going to the cinema/theatre/café/restaurant, attending religious services, visiting people, associative activities, etc.)** | 4/26 (15.4) | 2/26 (7.7) | **20/26 (76.9)** |
| 1. **Having a sense of direction and purpose in life** | **18/26 (69.2)** | 2/26 (7.7) | 6/26 (23.1) |
| 1. **Level of resilience** | **14/26 (53.8)** | 4/26 (15.4) | 8/26 (30.8) |
| ^a^ Criteria considered ‘not relevant’ over the e-Delphi rounds.  Note 1: In the first round, experts proposed eight additional criteria (criteria 81 to 88), which were subsequently incorporated into the second-round questionnaire. However, these additional criteria were not reviewed regarding the type of assessment, as this process was exclusive to the initial round.  Note 2: Results highlighted in bold indicate the criteria for which at least 50% of the experts positioned themselves in favour of that specific type of assessment. | | | |
